# Using minor variant genomes and machine learning to study the genome biology of SARS-CoV-2 over time

**DOI:** 10.1093/nar/gkaf077

**Published:** 2025-02-19

**Authors:** Xiaofeng Dong, David A Matthews, Giulia Gallo, Alistair Darby, I’ah Donovan-Banfield, Hannah Goldswain, Tracy MacGill, Todd Myers, Robert Orr, Dalan Bailey, Miles W Carroll, Julian A Hiscox

**Affiliations:** Institute of Infection, Veterinary and Ecological Sciences, Faculty of Health and Life Sciences, University of Liverpool, Liverpool, L3 5RF, United Kingdom; School of Cellular and Molecular Medicine, University of Bristol, Bristol, BS8 1TD, United Kingdom; The Pirbright Institute, Pirbright, Woking, GU24 0NF, United Kingdom; Institute of Infection, Veterinary and Ecological Sciences, Faculty of Health and Life Sciences, University of Liverpool, Liverpool, L3 5RF, United Kingdom; Institute of Infection, Veterinary and Ecological Sciences, Faculty of Health and Life Sciences, University of Liverpool, Liverpool, L3 5RF, United Kingdom; NIHR Health Protection Research Unit in Emerging and Zoonotic Infections, L69 7BE, Liverpool, United Kingdom; Institute of Infection, Veterinary and Ecological Sciences, Faculty of Health and Life Sciences, University of Liverpool, Liverpool, L3 5RF, United Kingdom; Office of Counterterrorism and Emerging Threats, U.S. Food and Drug Administration, Silver Spring, MD 20993-0002, United States; Office of Counterterrorism and Emerging Threats, U.S. Food and Drug Administration, Silver Spring, MD 20993-0002, United States; Office of Counterterrorism and Emerging Threats, U.S. Food and Drug Administration, Silver Spring, MD 20993-0002, United States; The Pirbright Institute, Pirbright, Woking, GU24 0NF, United Kingdom; NIHR Health Protection Research Unit in Emerging and Zoonotic Infections, L69 7BE, Liverpool, United Kingdom; Wellcome Centre for Human Genetics, Nuffield Department of Medicine, Oxford University, Oxford, OX3 7BN, United Kingdom; Pandemic Sciences Institute, Nuffield Department of Medicine, Oxford University, Oxford, OX3 7BN, United Kingdom; Institute of Infection, Veterinary and Ecological Sciences, Faculty of Health and Life Sciences, University of Liverpool, Liverpool, L3 5RF, United Kingdom; NIHR Health Protection Research Unit in Emerging and Zoonotic Infections, L69 7BE, Liverpool, United Kingdom; A*STAR Infectious Diseases Labs (ID Labs), A*STAR, Singapore, 138648, Singapore

## Abstract

In infected individuals, viruses are present as a population consisting of dominant and minor variant genomes. Most databases contain information on the dominant genome sequence. Since the emergence of SARS-CoV-2 in late 2019, variants have been selected that are more transmissible and capable of partial immune escape. Currently, models for projecting the evolution of SARS-CoV-2 are based on using dominant genome sequences to forecast whether a known mutation will be prevalent in the future. However, novel variants of SARS-CoV-2 (and other viruses) are driven by evolutionary pressure acting on minor variant genomes, which then become dominant and form a potential next wave of infection. In this study, sequencing data from 96 209 patients, sampled over a 3-year period, were used to analyse patterns of minor variant genomes. These data were used to develop unsupervised machine learning clusters to identify amino acids that had a greater potential for mutation than others in the Spike protein. Being able to identify amino acids that may be present in future variants would better inform the design of longer-lived medical countermeasures and allow a risk-based evaluation of viral properties, including assessment of transmissibility and immune escape, thus providing candidates with early warning signals for when a new variant of SARS-CoV-2 emerges.

## Graphical Abstract

**Graphical Abstract:**
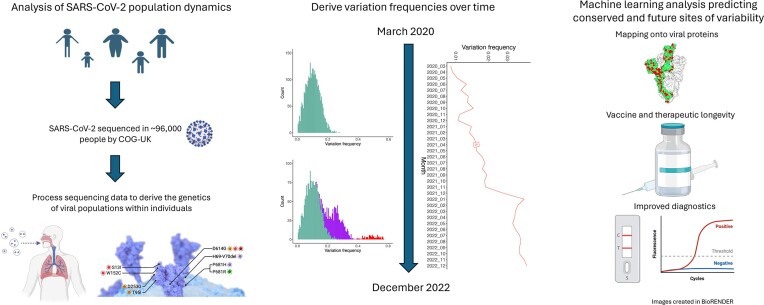

## Introduction

The mutational landscape of SARS-CoV-2 can vary during infection within an individual at both the dominant viral genome sequence and minor variant genomes [1, 2]. The minor variant genomes can contain single nucleotide polymorphisms (SNPs), insertions, and deletions that represent genetic diversity underneath the dominant genome sequence. As a simple example, a viral protein encoded by the dominant genome will have an A at a particular position with a frequency of 80% with minor variant genomes encoding a P (15%) and an R (5%) at the same position. Information on the dominant and minor variant genomes can be derived from sequencing data (e.g. [3–5]). After spillovers from the intermediate animal host in the Huanan Seafood Wholesale Market [6, 7], subsequent human-to-human infection has resulted in SARS-CoV-2 genomic diversification. Mutations have arisen through genomic changes from virus- and host-derived SNPs (e.g. induced by the ADAR and APOBEC family members) [8, 9], homologous and heterologous recombination events [10], and insertions and deletions (indels) [1]. The first major changes in the SARS-CoV-2 genome in the human population that had a noticeable phenotypic effect were the D614G substitution in the Spike glycoprotein (Spike) accompanied by the P323L substitution in the viral RNA-dependent RNA polymerase (NSP12). These changes were associated with increased transmissibility, replication, and fitness (e.g. [4, 11, 12]).

Dominant and minor variant genomes exist in humans and animals infected with SARS-CoV-2 [13, 14] (Supplementary Fig. S1A). These genomes will have both synonymous (non-coding) and non-synonymous (coding) differences. Minor variant genomes may become dominant when the proteins they encode or their replication/transcription or structural properties of their genomes confer an advantage under evolutionary force or through chance infection of an individual with a virus containing that particular minor variant genome, thus imposing an effective population bottleneck. If amino acid substitutions are neutral, the change in frequency of an amino acid may be due to non-directional stochastic evolutionary processes. If amino acid substitutions are non-neutral, the amino acid frequencies can be decided by directional positive and purifying selection. Either/both positive selection or/and stochastic evolutionary processes could result in a change in amino acid frequency.

Since the start of the COVID-19 pandemic, different dominant viral genome sequences and non-synonymous changes appear to rise and fall in the SARS-CoV-2 global sequence databases [15, 16]. Genetic change has resulted in the sequential dominance of several variants of concerns (VoCs) associated with waves of high case numbers throughout the pandemic. With time, VoCs have emerged with examples representing divergence from circulating strains, with many novel mutations having the appearance of occurring at once, such as with the emergence of Alpha [10] and Omicron [17]. One hypothesis is that these divergent variants may stem from persistent infection in immune-deficient hosts [10, 18]. These changes can occur both within coding regions (such as the P323L substitution in NSP12 [4] and the D614G substitution in Spike [11]) and outside of coding regions, which then can impact the regulation of viral transcription [19], e.g. with the regulation of the expression of viral innate immune antagonists in the Alpha VoC [20].

Databases, such as GISAID and the NCBI SARS-CoV-2 Data Hub, have collected tens of millions of SARS-CoV-2 dominant genomes since the start of the pandemic. The dominant genomes have been widely used for phylogenetic studies [21–24] and calling mutations of the virus genomes [25–28]. However, dominant genomes and major nucleotide/amino acid changes only display the results of evolutionary pressure. Amino acid variation frequencies, especially the frequencies of minor amino acid variations, can reveal the action of evolutionary pressure on SARS-CoV-2 and the emergence of minor genomic variants as dominant sequences. Recent research has employed a statistical modelling approach to predict the future prevalence of current mutations [16] and variants [29] based on dominant genomes subsampled from the GISAID database. Machine learning models have also been developed using the dominant genomes to predict the immune escape potential of emerging variants [30, 31] or combinations of current mutations that may predominate in the future [32]. The statistical and machine learning models are limited to working only for the variants of dominant genomes that have emerged and been detected. In this study, artificial intelligence/machine learning (AI/ML) was used to investigate the evolutionary patterns of amino acid changes (variation frequencies) at a population level, independent of the dominant viral genome sequences, and used to identify the conserved and future mutable amino acid sites in Spike. Avoiding these mutable amino acid sites in Spike could enhance the development and performance of medical countermeasures for SARS-CoV-2.

## Materials and methods

### Data collection

During the COVID-19 pandemic, COG-UK (COVID-19 Genomics UK Consortium) collected and sequenced SARS-CoV-2 genomes in the UK. To date, this project has sequenced over 2 600 000 SARS-CoV-2 genomes. The sequencing data are currently freely available at the NCBI SRA database. In this study, SARS-CoV-2 sequence data were sampled daily from different sites in the UK. To ensure consistency, sequence data were selected for viral genomes that had been amplified using ARTIC primers [33] and sequenced in paired-end mode on the Illumina NovaSeq 6000 platform. We randomly selected 100 samples per day (if available) with a fastq size >100 Mb, spanning from March 2020 to December 2022. A total of 96 558 samples were used for analysis (Supplementary Table S1). After removing the samples with a consensus genome covering <90% of the coding region, 96 209 samples were used for amino acid variation analysis (Supplementary Table S2). The fastq data were trimmed and cleaned by COG-UK. Therefore, we did not apply any further processing to these paired-end FASTQ files after retrieving them from the NCBI SRA database using ‘fastq-dump’ in the SRA-Toolkit.

### Amino acid variation analysis

The minor variations of amino acids encoded by the genes of the virus were called based on our previous methodologies [3, 4]. The paired-end fastq files obtained from the NCBI SRA database were then mapped on a known SARS-CoV-2 genome (Wuhan-Hu-1, GenBank sequence accession: NC_045512.2) using Bowtie2 v2.3.5.1 [34] by setting the options to parameters ‘--local -X 500 --no-mixed’, followed by SAM file to BAM file conversion, sorting, and removal of the reads with a mapping quality score below 11, not in pair, and not primary and supplementary alignment using SAMtools v1.9 [35]. Bamclipper (v1.0.0) [36] was used to trim the ARTIC primer sequences on the mapped reads within the BAM file. The reads without ARTIC primer sequences were also excluded in the further analysis. The resultant BAM file was processed by Quasirecomb v1.2 [37] to generate read coverage of each nucleotide site and a phred-weighted table of nucleotide frequencies. The phred-weighted table was parsed with a custom Perl script to generate a dominant genome sequence as per our previous description [38]. The dominant genome sequence was then used as a template in the second round of mapping to generate a BAM file and consensus genome. Only samples with a consensus genome covering 90% of the coding region were further processed. This BAM file was then processed by the diversiutils script in DiversiTools (http://josephhughes.github.io/DiversiTools/) with the ‘-orfs’ function to generate the sequencing coverage (number of codons in the mapped sequencing reads) and amino acid variation caused by nucleotide deviations in the mapped sequencing reads at each amino acid position in the protein. Then, the number of amino acid changes in the mapped sequencing reads at each site was compared to the reference SARS-CoV-2 genome (Wuhan-Hu-1, GenBank sequence accession: NC_045512.2). In order to distinguish low-frequency mutations from Illumina sequence errors, the diversiutils script used the Illumina quality scores to calculate a *P*-value for each mutation at each nucleotide site [39]. The subsequent amino acid change was then filtered based on the *P*-value (<.05) to remove the low-frequency mutations caused by Illumina sequence errors. The amino acid site variation frequency was calculated as the ratio between the number of amino acid changes and the coverage at each amino acid site. This pipeline has been packaged into an efficient and user-friendly tool called AioMinor (https://github.com/Hiscox-lab/AioMinor) for processing large datasets in this project.

### Shapiro–Wilk test and skewness

The normality of amino acid variation frequencies at each site in the samples (∼3000) collected in a month was tested using the Shapiro–Wilk test [40]. Only the amino acid sites with a sequencing coverage (number of the codon of amino acid in the mapped sequencing reads) >5 were included in the calculation. The Shapiro–Wilk test was performed using the Perl module ‘Statistics::Normality’, and the *W*value was reported as a measure of the normality of the test data [40]. The asymmetry of the amino acid variation frequency distribution at each site in each month was measured by skewness [41], which was calculated by the Perl module ‘Math::Stat’.

### Unsupervised machine learning

Unsupervised machine learning was performed with R language v4.1.3 [42]. The *W* values of each amino acid site in the selected months were combined into a data matrix. The Principal Component Analysis (PCA) method was then applied to the data matrix to create new variables using the pca function in the PCAtools package (v2.6.0). The parameters ‘removeVar = 0.1, center = T, scale = T’ in the pca function were used to standardize the data and remove 10% of variables based on low variance. Parallel analysis was carried out with the parallelPCA function from the PCAtools package to determine the number of principal components to retain. The retained principal components were used for clustering analysis using the pam algorithm from the cluster package (v2.1.4) with default settings. The optimal number of clusters in the pam algorithm was determined using the wss statistic from the fviz_nbclust function in the factoextra package (v1.0.7).

## Results

### Genetic variation in SARS-CoV-2 increased with time

To understand the emergence of minor variant genomes and derive amino acid variation frequencies, sequence data gathered by COG-UK were analysed. Sequences produced by the ARTIC amplification method and sequenced by Illumina NovaSeq 6000 were selected. In total, 96 559 SARS-CoV-2 consensus genomes were derived, together with the associated minor variant genomic information (representing ∼100 genomes per day for nearly 3 years, starting in March 2020 up to 31st December 2022). Such large sample sets would provide sufficient information to depict the evolution of SARS-CoV-2 genomes from the start of the pandemic to recent times and allow equivalence and standardization of sequencing approaches in providing data and evaluation for and of unsupervised machine learning approaches.

**Figure 1.**
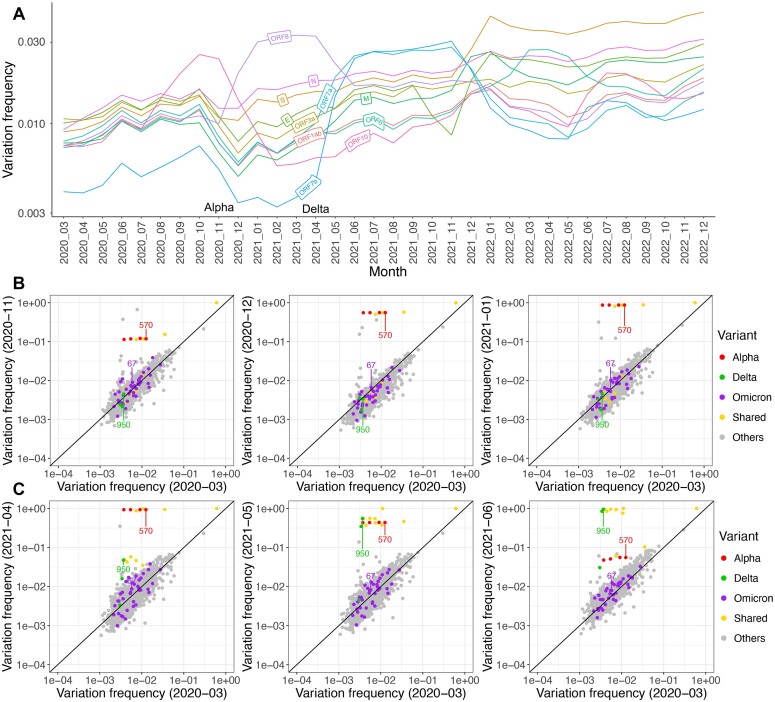
(**A**) Diversity of minor genomic variants in SARS-CoV-2 from March 2020 to December 2022 was determined by sampling 100 genomes per day. Individual proteins are indicated both by coloured lines and labelling. Diversity increased from the start of the pandemic. Scatter plots showing the amino acid variation in Spike for (**B**) November 2020, December 2020, and January 2021 (emergence of Alpha) compared to March 2020 and (**C**) April, May, and June 2021 (emergence of Delta). The straight line indicates no change in the amino acid variation frequency between the 2 months being compared. Substitutions associated with VoCs are coloured according to the key. As a substitution becomes fixed, the amino acid moves up. The location of one amino acid of each VoC is labelled in panels (B) and (C).

The nucleotide sequences were *in silico* translated into amino acid sequences. The average variation frequencies of each amino acid were calculated to investigate the pattern of substitution in SARS-CoV-2 proteins over the nearly 3-year period sampled. We found the presence of artificial low-frequency genetic variants due to errors acquired during amplicon generation and sequencing (Supplementary Fig. S1B and C; see more details in ‘Supplementary data’). To mitigate this effect, the average variation frequencies of amino acids at the same site in different months were compared with each other to reveal differences in frequencies of amino acid changes, instead of comparing different sites to each other. The average variation frequencies of amino acid sites in each viral protein were compared in monthly intervals from March 2020 to December 2022 (Fig. 1A). These data revealed that the majority of viral proteins (except ORF7a, ORF7b, ORF8, and ORF10) had a similar pattern of increasing diversity from the start of the pandemic, until a slight decrease associated with the emergence and rapid spread of the Alpha variant in October 2020 and then subsequent increasing diversity at the level of minor variant genomes between December 2020 and January 2021 (Fig. 1A).

### Real-time emergence of amino acid substitutions associated with VoCs in the UK

**Figure 2.**
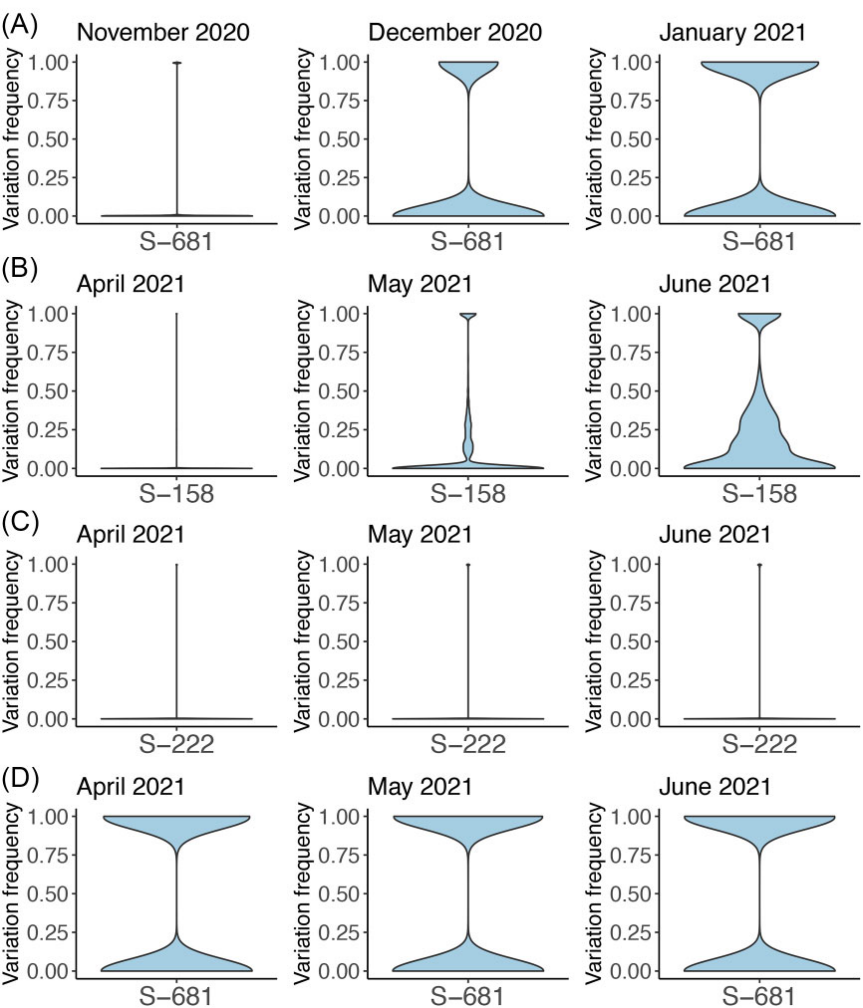
Exemplar analysis showing the transition of (**A**) S681 in Alpha and for (**B**) S158, (**C**) S222, and (**D**) S681 in Delta in the Spike protein over time in the minor genomic variants (full data shown in Supplementary Fig. S3). The width of the violin plot indicates the number of samples with the frequency on the *y*-axis. Samples with a variation frequency = 1.00 indicate a complete transition of the mutation. Not all substitutions associated with each VoC have an identical pattern of becoming fixed.

Two different analyses were used to study the emergence of different amino acid substitutions and their frequencies. The first analysis visualized data as dot plots (Fig. 1B and C, Supplementary Fig. S2, and Supplementary Table S3). These showed the monthly change in average variation at each amino acid position in Spike compared to March 2020 (where there was little variation, and therefore this was used as a fixed point of comparison to benchmark subsequent changes) or October (16th to 31st) 2021 (when ARTIC primer sets used in COG-UK sequencing changed from version 3 (B) to version 4). The second analysis visualized data as violin plots to show the frequency of amino acid substitutions at different sites in individual people sampled as part of this study at monthly intervals (Fig. 2 and Supplementary Fig. S3).

The monthly average variation frequency of each amino acid site in SARS-CoV-2 from April 2020 to October (1st to 15th) 2021 was compared to that of March 2020 (Supplementary Fig. S4—animation of variation for each protein and Supplementary Table S3). Because the ARTIC primers were updated from version 3 to version 4 from 15th October 2021 (to mitigate amplicon dropouts caused by the emergence of the Delta variant), the monthly average variation frequency from November 2021 to December 2022 was compared to that of October (16th to 31st) 2021. We analysed the evolutionary process of the emergence and development of substitutions in the virus with the variation frequency of each amino acid site. As a proportion of the viral genome/proteome, the data indicated that Spike had the greatest number of substitutions, likely reflecting the fact that Spike is under strong evolutionary pressure due to the differing and often interlocked functions of the protein—including cell entry, transmission, and host immunity [43–45] (Supplementary Fig. S4 and Supplementary Table S3) [43, 44, 46]. As an example, for the average variation frequencies of amino acid sites in Spike in April 2020, they were like those in March and showed little variability (Supplementary Fig. S2A—with all data points revolving around the 1:1 line). However, at this time, the first major change, D614G, associated with the emergence of a new variant with increased transmission could be observed (yellow-coloured at the top right corner, Supplementary Fig. S2A, and Supplementary Table S3). This had an average variation frequency of ∼1.0 in March and April 2020 [4]. The D614G substitution was then maintained in all subsequent variants in this study without a decline in the variation frequency over the 3-year period sampled (Supplementary Fig. S2).

Two VoCs (Alpha and Delta) were prevalent in the UK before the introduction and evolution of the Omicron VoCs (Supplementary Fig. S2 and Supplementary Table S3). Analysis indicated that amino acid substitutions associated with the Alpha VoC were emerging from October 2020 as minor variant genomes (Supplementary Fig. S2 and Supplementary Table S3). By November and December 2020, these substitutions were becoming more prevalent and became dominant in January 2021 (Fig. 1B, Supplementary Fig. S2, and Supplementary Table S3). Afterwards, substitutions associated with the Delta VoC emerged in April 2021 (Fig. 1C, Supplementary Fig. S2, and Supplementary Table S3) and were fixed by June 2021, while Alpha and Delta were coexisting in May 2021 [4].

The first viral genome/proteome change associated with a significant change in viral properties (the P323L substitution in NSP12 and D614G substitution in Spike) took 3 months to increase in frequency in the UK (and worldwide) from minor variant genomes to become the dominant sequence [4]. This was evidenced during the containment phase in the UK (a total of 317 samples sequenced over 3 months) and worldwide (782 sequences used), when in February 2020, sequencing data of cases of COVID-19 indicated that P323 and D614 were still dominant. Moving into March and April 2020, P323L and D614G were roughly equal in frequency. However, by May 2020, L323 and G614 were dominant [4]. Most of the amino acid substitutions associated with the Alpha and Delta VoCs followed a similar pattern of time taken to emerge (at least in the UK) (Supplementary Fig. S3). In that, substitutions associated with these VoCs took around 3 months to emerge from minor genomic variants into dominant sequence, and there was a period of transition where the wild-type and new variants coexisted at a dominant level in a large proportion of the infected human population. For instance, in November 2020 in the UK, the N501Y substitution in Spike (associated with the Alpha VoC) started with Y501 in minor variant genomes in the infected human population, and then in December 2020 genomes with both N501 and Y501 were present at a dominant level in a large proportion of the infected human population. However, by January 2021 in the UK, Y501 was dominant in most of the human population (Supplementary Fig. S3A). However, some substitutions showed a different pattern of emergence; e.g. the P681H in Spike from the Alpha VoC (Fig. 2A and Supplementary Fig. S3A) and R158G, A222V, and P681R in Spike from the Delta VoC (Fig. 2B–D, and Supplementary Fig. S3B).

### Evolutionary patterns and substitution from the start of the pandemic

**Figure 3.**
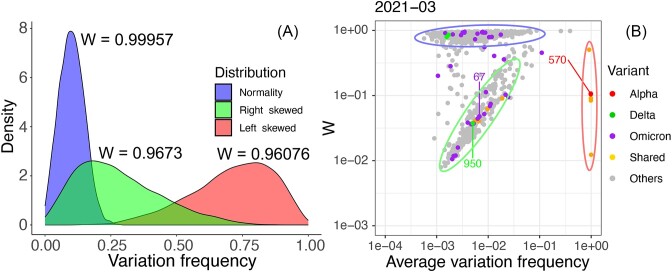
(**A**) Examples of approximate normal distribution, right-skewed distribution, and left-skewed distribution of the variation (*W* values for the distributions are shown above the curves) in a particular amino acid in Spike. (**B**) *W* value plotted against the average variation frequency for each amino site in each month showing an exemplar month of March 2021 when the Alpha variant was fixed in the UK and Delta was introduced. Amino acid sites were clustered based on the *W* values and highlighted with the same colours corresponding to panel (A). Each dot represents an individual amino acid site in Spike. Substitutions associated with particular VoCs are indicated by the colour in the key. The location of one amino acid of each VoCs is labelled in panel (B).

To investigate whether we could rank potential amino acids in Spike that would be subject to evolutionary pressure, the *W* value was used to measure the evolutionary pressure on amino acids in these samples (for a more in-depth discussion of the *W*value, see Supplementary data). The *W* value is also referred to as the Shapiro–Wilk statistic *W* (*W* for Wilk), and its range is 0–1 to measure the normality of a distribution of data [40]. *W* values close to 1 indicate the normality of the data, while a value close to zero indicates the non-normality of the data [47]. In this study, the *W* value was used to measure the evolutionary force acting on each amino acid site in Spike per month for 34 months. If evolutionary pressure acts on an amino acid site, this will change the distribution of variation frequencies, and therefore the *W* value will change and drop away from 1. To illustrate this, simulated data (Fig. 3A) were controlled by two parameters, the variation frequency and the skewness, that are used to derive the *W* value.

Simulated data were used to demonstrate how evolutionary pressure affected the distribution of the variation frequencies and altered the *W* value at an amino acid position in Spike. The variation frequency at a hypothetical site in Spike was simulated in 3000 patients to demonstrate the effects of sequencing error rate and evolutionary pressure on the *W* value. If we assume that this site was not subject to any evolutionary pressure, the observed variations at this position would be a result of errors during sequencing. In that case, the frequency of variation at an amino acid site in monthly samples would be predicted to exhibit symmetry around the mean and display an approximate normal distribution and have a high *W* value tended towards 1 (Fig. 3A, blue distribution) (see more details in Supplementary data). If this site was subsequently experiencing evolutionary pressure, this would raise the proportion of substituted amino acid(s) at this site in minor genomic variants or even to dominant genomes in some individuals, generating a right-skewed distribution of the data and therefore a lower *W* value (Fig. 3A, green distribution). Stronger evolutionary pressure would increase the proportion of substituted amino acid(s) to a dominant level in most of the individuals at the site (substituted amino acids become fixed in the population), and then this will also result in a low *W* value, but in this case the data have a left-skewed distribution (Fig. 3A, red distribution). Therefore, a *W* value that tends towards 1 could be an indicator of a conserved amino acid site.

We investigated whether the real data of amino acid substitutions in Spike and their frequency could fit the models of skewed distribution and corresponding *W* values described above. Supplementary Fig. S5 shows the direction of skew and *W* value of the variation frequency at each amino acid site of the Spike protein per month for 34 months. The substituted amino acids of the Alpha, Delta, and Omicron VoCs showed right-skewed distributions and low *W* values before being fixed in the population in January 2021, June 2021, and January 2022, respectively (fitting the model shown in Fig. 3A, green distribution). After becoming fixed in the population, substituted amino acids of the Alpha, Delta, and Omicron VoCs showed left-skewed distributions and low *W* values in January 2021, June 2021, and January 2022, respectively (see Supplementary Fig. S5 and Supplementary Table S4) (fitting the model shown in Fig. 3A with the red distribution).

We next examined the patterns of variation of amino acid substitutions over the 34-month period by considering the interaction between the *W* value and the variation frequency using the real-world data. Consideration of these parameters would provide information on whether similar *W* values and variation frequencies in amino acids can cluster together and how these change with time. To illustrate this concept and provide proof of principle before describing the full analysis, we derived the *W* value and average variation frequency of each amino acid site in Spike for March 2021 (presented as a dot plot—Fig. 3B), which indicated that amino acids were grouped into three clusters. The first cluster (Fig. 3B, amino acid sites in blue ellipsoid) was amino acids with a *W* value tending towards 1, which suggested little evolutionary pressure. These data generally fitted the model shown in Fig. 3A by the blue distribution. The second cluster consisted of amino acids with a low *W* value and a slightly increased average variation frequency (Fig. 3B, amino acid sites in green ellipsoid; the increase is better observed in the animation in Supplementary Fig. S6 and Supplementary Table S5). These data generally fitted the model presented in Fig. 3A by the green distribution. The third cluster comprised of amino acids with low *W* values and an average variation frequency around 1 (Fig. 3B, amino acid sites in red ellipsoid). This fitted the model shown in Fig. 3A with the red distribution.

The relationship between the *W* value and the average variation frequency was then visualized for each amino site in Spike for 34 months. In general, two separated clusters of amino acid variation in Spike were observed throughout the months analysed, similar to the snapshot of data discussed earlier (Supplementary Figs S6 and S7, Fig. 3B, and Supplementary Table S5). Some amino acids in Spike had high *W* values close to 1 throughout all 3 years sampled, some remained in the low *W* value cluster, and some shifted between high and low W value clusters (Supplementary Figs S6 and S7 and Supplementary Table S5). Interestingly, we found that amino acid sites associated with the VoCs (Alpha, Delta, and Omicron) remained in the low *W* value cluster or shifted between the two clusters (under evolutionary pressure) from the start of the COVID-19 pandemic (Supplementary Figs S6 and S7 and Supplementary Table S5). These amino acids would travel through the cluster associated with a lower *W* value (as the minor variant became a high proportion in the population sampled) before they become fixed in the population (Supplementary Figs S6 and S7, Fig. 3B—in this snapshot for the Alpha VoC, and Supplementary Table S5).

We note that our approach was focused and optimized to identify non-synonymous changes. However, we also examined nucleotide mutations in the transcription regulatory sequence (TRS) region of each sub-genome, as these changes could potentially alter the expression of viral sub-genomic mRNAs and hence proteins. Although changes in *W* values at individual nucleotide sites were observed, almost no mutations at these sites became fixed in the population (Supplementary Fig. S8). The only exception was a change in the TRS of ORF7b (C to U at position 28271), ORF8 (C to U at position 27889), and N (A to U at position 28271).

### Identifying future mutable sites in SARS-CoV-2 Spike

**Figure 4.**
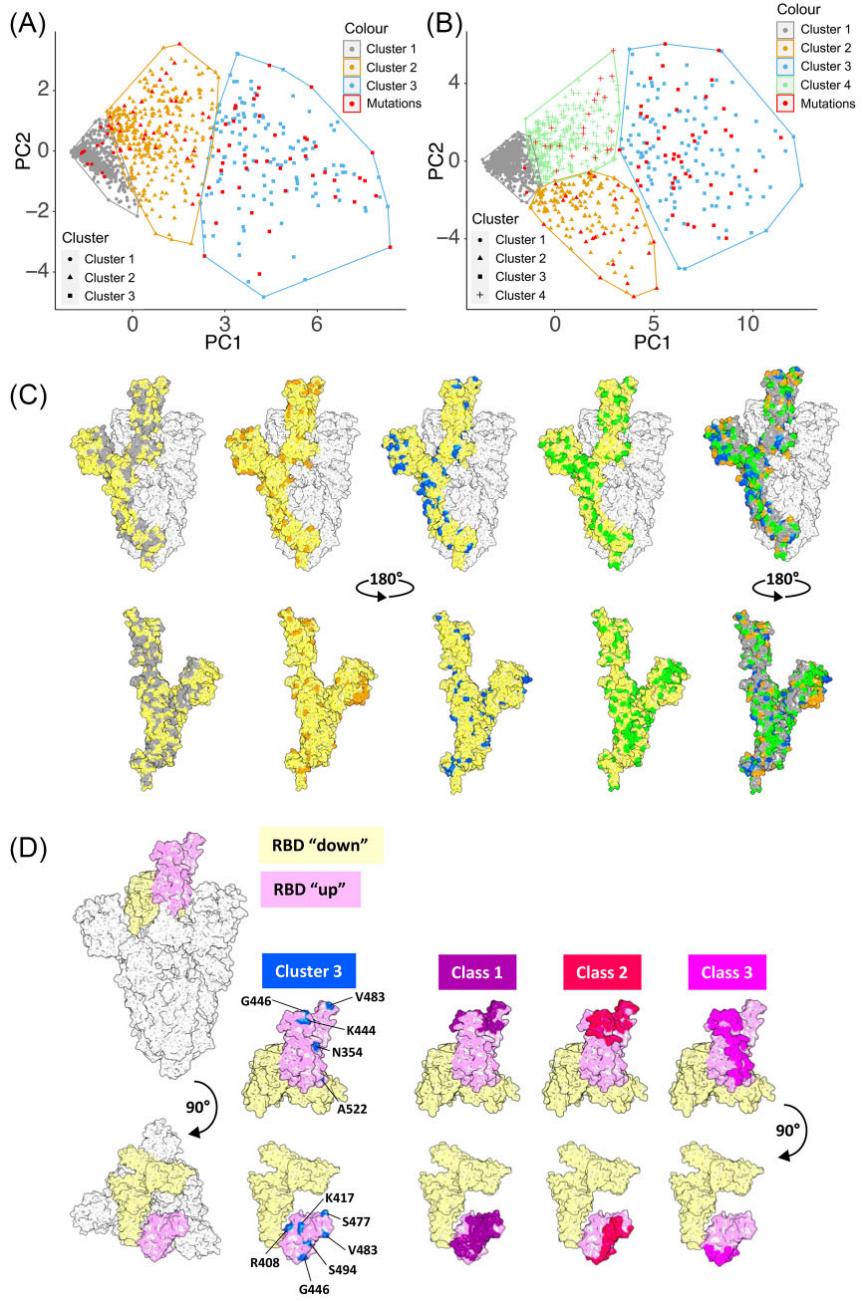
Scatter plots of pam clusters derived by AI/ML with colouring and shaping each data point according to its cluster assignment for (**A**) March 2020 to May 2020 (pam12) and (**B**) March 2020 to February 2021 (pam34). (**C**) Diagram of the Spike protein showing major functional regions with the different clusters indicated from the pam34 clusters. The structure of Spike was modelled in PyMOL, and an individual monomer is shown in yellow as part of the trimer. Amino acids associated with the clusters identified in pam34 are shown in their respective colours. (**D**) Model of the Spike protein showing exemplar locations of specific sites identified in cluster 3 and how these map to part of the receptor binding domain and interact with different classes of antibodies.

The determination for a *W* value for each amino acid in Spike in a large dataset, given the background context of evolutionary forces acting on a population, may provide an indicator for whether that site would be subject to future evolution in an individual (or population). To identify which sites in Spike may be subject to future evolutionary pressure, an unsupervised machine learning technique was used that identified models (patterns, structures, or relationships) in a dataset without any prior knowledge (see more details in Supplementary data). The *K*-medoid clustering (pam) algorithm was used to cluster amino acid sites based on the *W* values from March 2020 to May 2020 for 3 months (pam3), March 2020 to August 2020 for 6 months (pam6), March 2020 to November 2020 for 9 months (pam9), March 2020 to February 2021 for 12 months (pam12), and March 2020 to December 2022 for 34 months (pam34) (Fig. 4A and B and Supplementary Fig. S9).

To evaluate the performance of the models to cluster amino acids with similar *W* values and to investigate whether these were related to potential variability, we examine substitutions in Spike associated with World Health Organization VoCs (https://covariants.org, updated on 26 April 2023, Supplementary Table S6), substitutions that were most frequent as tracked by https://www.bv-brc.org (updated on 12 January 2023) in frequencies of 0.001 and 0.005 (Supplementary Table S7), and substitutions that appeared in any active Pango lineage (collected by https://cov-lineages.org, extracted on 10 June 2023, Supplementary Table S8). In general, the pam clusters performed better when the models were calculated using *W* values from more months (Fig. 4A and B, Supplementary Fig. S9, and Table 1). Amino acid sites within the cluster exhibiting the highest *W* values were potentially conserved, while substitutions were more likely to occur on those amino acids within other clusters with lower *W* values. In all the developed pams, only a small number of tested substitution sites were found in cluster 1, which had the highest *W* values. Most of the tested substitution sites were found in the clusters with the lower *W* values (Table 1). Using the pam12 clusters, ∼13% of the substitution sites of interest were found in cluster 1, while ∼87% were found in the other clusters (Table 1). The pam12 clusters identified 27 out of 39 (69.2%) Omicron-specific mutation sites that emerged since late 2021 (Supplementary Table S6). Similarly, the pam34 model identified 98% of the substitution sites of interest (Table 1). Both the pam12 and pam34 models also demonstrated excellent performance in clustering the substitution sites that occurred most frequently (0.001 and 0.005) (Table 1).

**Table 1.** Summary of mutations tested by the cluster models

Months	Cluster	Average *W* values	Number of AA sites in cluster	Mutations (VoCs)	Mutations (freq > 0.005)	Mutations (freq > 0.001)	Mutations (all active Pango lineage)	Mutations (active Pango lineage after Match 2021)
1–3 (pam3)	Cluster 1	0.75308	958	41 (44.57%)	35 (48.61%)	56 (36.60%)	117 (55.19%)	78 (57.35%)
	Cluster 2	0.16662	69	21 (22.83%)	15 (20.83%)	40 (26.14%)	28 (13.21%)	15 (11.03%)
	Cluster 3	0.43323	121	15 (16.30%)	11 (15.28%)	23 (15.03%)	38 (17.92%)	26 (19.12%)
	Cluster 4	0.42764	126	15 (16.30%)	11 (15.28%)	34 (22.22%)	29 (13.68%)	17 (12.50%)
1–6 (pam6)	Cluster 1	0.8225	691	25 (27.17%)	21 (29.17%)	27 (17.65%)	67 (31.60%)	43 (31.62%)
	Cluster 2	0.62296	337	20 (21.74%)	16 (22.22%)	36 (23.53%)	53 (25.00%)	34 (25.00%)
	Cluster 3	0.27735	129	30 (32.61%)	17 (23.61%)	69 (45.10%)	55 (25.94%)	37 (27.21%)
	Cluster 4	0.53879	117	17 (18.48%)	18 (25.00%)	21 (13.73%)	37 (17.45%)	22 (16.18%)
1–9 (pam9)	Cluster 1	0.79224	656	17 (18.48%)	13 (18.06%)	19 (12.42%)	55 (25.94%)	40 (29.41%)
	Cluster 2	0.53973	167	25 (27.17%)	21 (29.17%)	41 (26.80%)	50 (23.58%)	28 (20.59%)
	Cluster 3	0.26138	136	38 (41.30%)	28 (38.89%)	76 (49.67%)	65 (30.66%)	38 (27.94%)
	Cluster 4	0.62497	315	12 (13.04%)	10 (13.89%)	17 (11.11%)	42 (19.81%)	30 (22.06%)
1–12 (pam12)	Cluster 1	0.77498	760	12 (13.04%)	12 (16.67%)	18 (11.76%)	49 (23.11%)	37 (27.21%)
	Cluster 2	0.57421	345	35 (38.04%)	29 (40.28%)	49 (32.03%)	87 (41.04%)	53 (38.97%)
	Cluster 3	0.27257	169	45 (48.91%)	31 (43.06%)	86 (56.21%)	76 (35.85%)	46 (33.82%)
1–34 (pam34)	Cluster 1	0.7657	636	2 (2.17%)	1 (1.39%)	3 (1.96%)	15 (7.08%)	13 (9.56%)
	Cluster 2	0.55819	169	25 (27.17%)	23 (31.94%)	35 (22.88%)	55 (25.94%)	27 (19.85%)
	Cluster 3	0.28194	166	44 (47.83%)	34 (47.22%)	89 (58.17%)	90 (42.45%)	57 (41.91%)
	Cluster 4	0.59439	303	21 (22.83%)	14 (19.44%)	26 (16.99%)	52 (24.53%)	39 (28.68%)

These models were also applied to cluster the 212 substitution sites in Spike found across all active Pango lineages (from a total of 826 lineages at the time of analysis), including 136 mutation sites (in 759 lineages) that were identified since March 2021 [3]. Although most of these Pango lineages were not prevalent and the substitutions that occurred at these sites were rare, the pam3, pam6, pam9 and pam12 models still identified ∼43%, 68%, 71%, and 73% of the 136 substitution sites, respectively (Table 1). To investigate whether one pam performed better than another, McNemar’s test was used to compare the classifiers between any two of these pams. This analysis found that pam12 was significantly better than pam3 (*P* = 3.29E−14) and pam6 (*P* = .01414) but was not significantly different from pam9 (*P* = .3268) (Supplementary Table S9). This suggested this method was effective at identifying (71%) future mutable sites from the first 9 months of data after the start of the pandemic.

The pam34 clusters were developed based on a large dataset (96 209 samples) collected over a 3-year period since the beginning of the pandemic. SARS-CoV-2 was subject to various evolutionary pressures present in these individuals (e.g. prior infection with SARS-CoV-2 generating partial immunity, potential immunity through vaccination, etc.). We speculate that, with the exception of a virological process such as recombination and treatment with antivirals, it is unlikely that many additional evolutionary pressures will act on the virus at a population level. Furthermore, no substitutions caused by the vaccine have been reported [48]. Therefore, we predict that the pam34 clusters would be expected to remain applicable for a significant period in the future. The sites were modelled onto the Spike protein using PyMOL (Fig. 4C and D) and, for example, identified a position V483 on the receptor binding domain that is associated with escape to monoclonal antibody therapy [49, 50] and G486 that is associated with a loss in neutralization [51]. This study identified sites in the Spike protein that are subject to change but also highlights those sites that remain conserved (Fig. 4C and D) and would logically inform part of a universal vaccine that would potentially offer some protection against severe disease caused by future variants.

## Discussion

As SARS-CoV-2 has transmitted between humans (and animals) over the past 4 years, there have been a number of genotype to phenotype changes, particularly focused on Spike [11, 12]. This protein has critical roles in viral entry into cells (through receptor recognition) and is a major target of the immune response. As such, many licensed vaccines (such as those based on the mRNA platform) encode Spike. Because of partial immunity achieved in the population (either through infection and/or through vaccination) and growth/transmission advantages [52] gained, different variants of the virus have emerged and are likely to emerge. This will result in changes in Spike and therefore require updating of vaccines, as we have seen with the introduction of bivalent vaccines [53, 54].

One of the lessons of the SARS-CoV-2 pandemic, and from ongoing outbreaks of EBOV and viruses such as HIV-1, is that the rapid pace of viral evolution can necessitate changes in medical countermeasures such as diagnostics, vaccines, and direct-acting antiviral therapies. For example, some monoclonal antibody therapies could not keep pace with the emergence of new variants of SARS-CoV-2 [55]. There have been waves of different dominant genome sequences of SARS-CoV-2—the most obvious ones being labelled as a VoC. These new variants can have different biological properties that provide a selective advantage, for example, caused by differences in Spike [11], the RNA-dependent RNA polymerase [4], or viral proteins involved in innate immune antagonism [20], or synergistic effects acting on different parts of the viral genome/proteome [56].

The ability to identify stable and unstable parts of a viral genome will aid in ensuring the longevity and more efficient design of medical countermeasures, e.g. identifying conserved immunodominant T-cell peptides to include as part of vaccine candidates [57], or building these potential changes into Spike and using appropriate assays (such as pseudoviruses) to risk assess them for phenotypes such as better receptor usage or neutralization [58]. This could provide early warning signals to risk assess potential new VoCs.

For SARS-CoV-2, statistical models have been developed to predict the future prevalence of current mutations [16] and variants [29]. Additionally, machine learning models have been designed to forecast the escape potential of emerging variants [30, 31] or combinations that may arise in the future [32]. Our model for the transmission of variant genomes [4] proposed that the distribution of minor variant genomes and the dominant viral genome sequence for SARS-CoV-2 is dependent on the evolutionary pressure and the time of post-infection at which a virus population is transmitted onwards to another individual [4]. Consequently, a significant number of variants and substitutions remain at low frequency. These minor variant genomes are not included in a sequencing database (such as GISAID) until they become dominant. Therefore, statistical and machine learning models can be constrained by using dominant genomes and not considering minor variant genomes.

To analyse patterns of amino acid substitutions in Spike, the variation frequency and *W* value of each amino acid position were derived over 34 months by sampling sequencing data for ∼300 patients per month using COG-UK data. We identified early substitution signals (in the minor variant genomes) at the start of the pandemic in the UK for substitutions at amino acid sites that would come to dominate the UK and global landscape either a year or several years later. These signals included substitutions that were subsequently the hallmarks of VoCs. This paralleled a previous analysis of sequencing data from patients associated with the Huanan Market, which identified substitutions characteristic of future VoCs that were represented in minor variant genomes at low frequency [59]. This is not to imply that these minor variant genomes were transmitted and their direct descendants became dominant sequences in the future, but it would at least highlight that genomic variability was potentially tolerated in these regions/at these sites—and this can be identified and measured. It is interesting to reflect that potentially some of the same building blocks of VoCs were geographically widely distributed, and therefore it was likely fortuitous circumstance that specific VoCs emerged in particular countries. For example, substitutions associated with Omicron were observed in the low *W* value cluster in the first 3 months of UK data analysed in 2020, although this variant only emerged in late 2021 with a proposed origin in Southern Africa [17] (Supplementary Figs S6 and S7).

**Figure 5.**
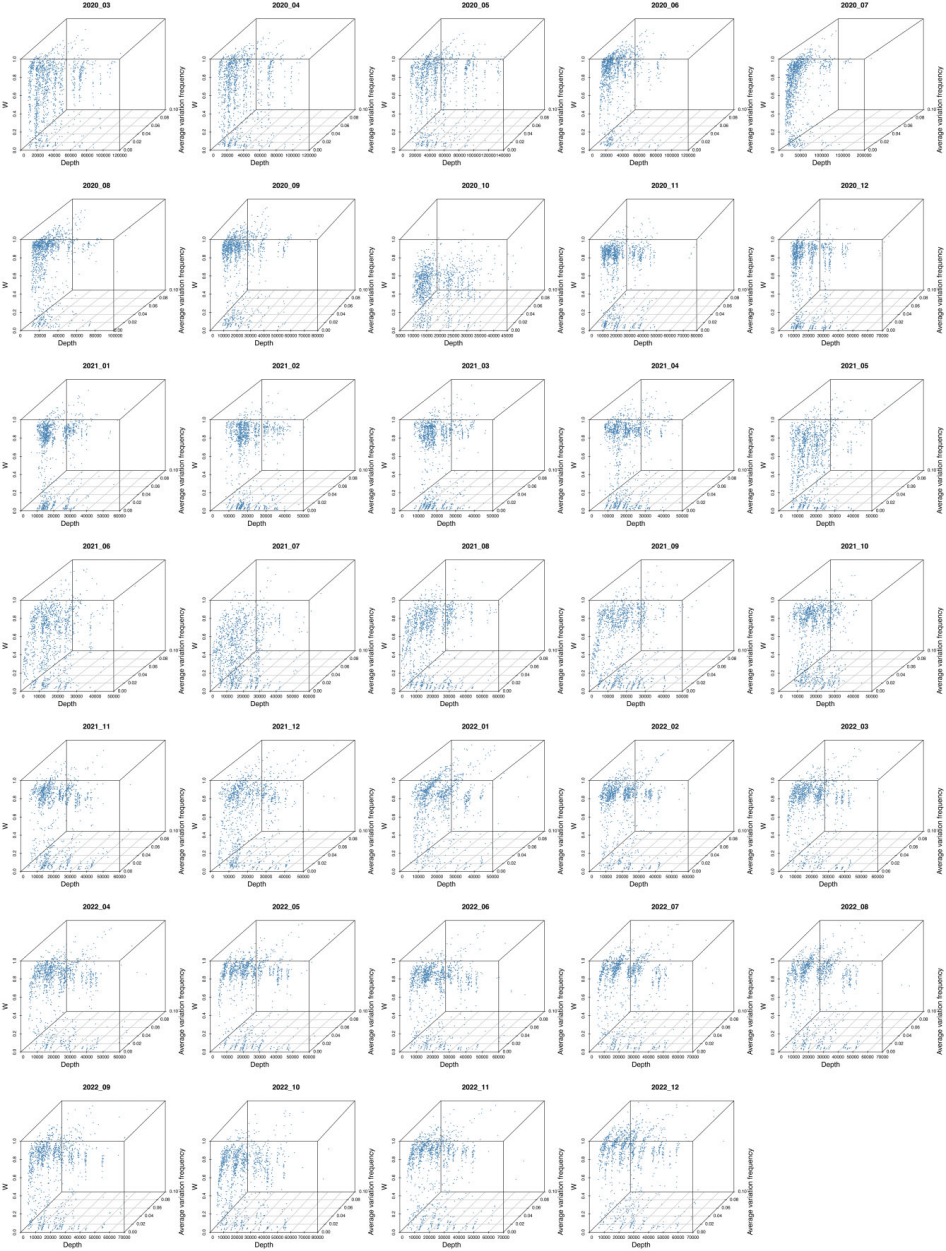
The average sequencing depth (from ∼3000 samples) of each amino acid site in Spike was plotted against their average variation frequency (from ∼3000 samples) and *W* value.

There were several advantages of using the*W* value to investigate evolutionary pressures in the SARS-CoV-2 genome using sequencing data as a basis. The *W* value overcame the periodicity of sequencing error (as seen in Supplementary Fig. S10 and more details in Supplementary data). The analysis also showed that the *W* value was not sensitive to sequencing read depth and low variation frequency (Fig. 5). We would note that our selection of sequencing data included consideration of sequence read depth, where we used data from patient samples where SARS-CoV-2 had been sequenced using amplicon approaches on an Illumina platform. The average sequencing depth for an amino acid site in Spike is ∼20 528.

A detailed pattern of the emergence of dominant sequences from minor variant genomes was derived over the 34-month sampling period. For Spike, amino acids tended to group into three distinct clusters: those that had high*W* values close to 1 throughout all ∼3 years sampled, those that remained in low *W* value cluster, and thoe shifted between high and low *W* value clusters (see more discussions for *W* value combined with Supplementary Figs S10 and S11 in Supplementary data). Our machine learning models were developed based on these early substitution signals, independent of the dominant viral genome sequences stored in global databases. Several machine learning models were compared based on the length of time of the training data. Both pam12 (using the first ∼12 months of sequencing data) and pam34 (using the ∼first 3 years of data) showed good performance in clustering amino acid sites of variability, suggesting that most evolutionary pressure that would occur in humans could be detected in the first 12 months of the pandemic. Therefore, analysing early sequencing data from viral outbreaks with machine learning may provide a way forward for the efficient development of longer-lived medical countermeasures at the start of a pandemic. This approach is likely to apply to viruses other than SARS-CoV-2 and would potentially guide sampling and sequencing strategies during a new pandemic.

## Supplementary Material

gkaf077_Supplemental_Files

## Data Availability

The SARS-CoV-2 genome sequencing data from COG-UK are currently freely available at the NCBI SRA database. Clusters in the models of pam3, pam6, pam9, pam12, and pam34 are available in Supplementary Table S10. The pipeline for deriving amino acid variation has been packaged into an efficient and user-friendly tool called AioMinor (https://github.com/Hiscox-lab/AioMinor, https://zenodo.org/records/14748828) for processing the large datasets in this project.

## Supplementary data

Supplementary data is available at NAR online.
