# Supplementary material for: Using minor variant genomes and machine learning to study the genome biology of SARS-CoV-2 over time
Source: Nucleic Acids Res. 2025 Feb 19;53(4):gkaf077. doi: 10.1093/nar/gkaf077 (PMC11838042; doi:10.1093/nar/gkaf077)
Supplement: gkaf077_Supplemental_Files [file gkaf077_supplemental_files.zip › Supplementary table and sound legends.docx]

**Supplementary** **table legends:**

Supplementary Table S1: The NCBI accession numbers for the 96,558 samples collected for initial analysis.

Supplementary Table S2: The NCBI accession numbers for the 96,209 samples after removing the samples with consensus genome covering less 90% of the coding in Dataset S4.

Supplementary Table S3: The original data for plotting Figure 1B and C, Supplementary Figure S2 and S4.

Supplementary Table S4: The original data for plotting Supplementary Figure S5.

Supplementary Table S5: The original data for plotting Figure 3B, Supplementary Figure S6 and S7.

Supplementary Table S6: The cluster results of each model (pam3, pam6, pam9, pam12 and pam34) for the substitutions in Spike associated with WHO VoCs.

Supplementary Table S7: The cluster results of each model (pam3, pam6, pam9, pam12 and pam34) for substitutions that are most frequent in frequencies of 0.001 and 0.005.

Supplementary Table S8: The cluster results of each model (pam3, pam6, pam9, pam12 and pam34) for the substitutions that appeared in any active Pango lineage.

Supplementary Table S9: P-values calculated by McNemar's test for comparing the classifiers between any two of these pams.

Supplementary Table S10: Clusters and Silhouette scores of each amino acid site of Spike in the models of pam3, pam6, pam9, pam12 and pam34.

**Supplementary sound legend**

The sound is for Supplementary Figure S6 (Mp4 of Dong’s sonata of SARS2 Spike), which is an interpretation of the W value by converting the data into music with Python (https://github.com/SYSTEMSounds/sonification-tutorials).
