# Supplementary material for: Using minor variant genomes and machine learning to study the genome biology of SARS-CoV-2 over time
Source: Nucleic Acids Res. 2025 Feb 19;53(4):gkaf077. doi: 10.1093/nar/gkaf077 (PMC11838042; doi:10.1093/nar/gkaf077)
Supplement: gkaf077_Supplemental_Files [file gkaf077_supplemental_files.zip › Supplementary_Figures.docx]

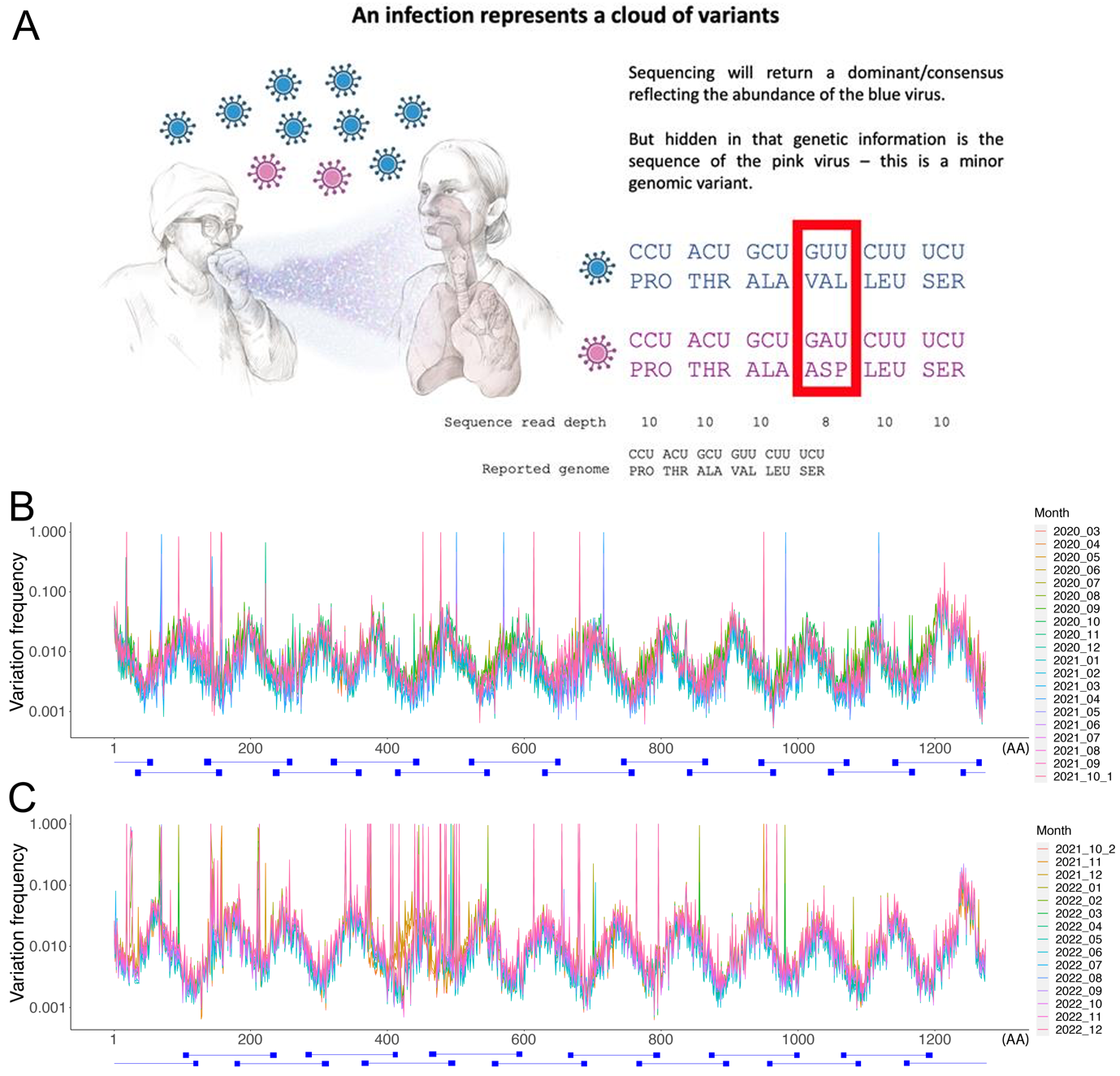


Supplementary Figure S1. (A) Diagram illustrating the concept of minor variant genomes (images of people from BioRender). Viruses exist as populations that are transmitted between individuals, and these can be sampled using swabs and then sequenced. The consensus/dominant viral sequence is normally reported but under that is information about minor variant genomes that can be at different ratios. For example, at an amino acid position a ration could be 51:49 if there are only two amino acid variants at that position or 85:10:5 if three amino acids variants at that position. We note that viral populations could be even more complex than this. (B) Average variation frequency of each amino acid site along the spike protein for each month from 1^st^ March 2020 to 15^th^ October 2021 (indicated to the right) and (C) 16^th^ October 2020 to 31th December 2022 (indicated to the right). ARTIC primers sets are indicated using blue boxes below the x-axis, and paired primers were linked by blue lines. 2021_10_1 strands for October (1^st^ to 15^th^) 2021 and 2021_10_2 strands for (16^th^ to 31^st^) 2021. Two different date ranges are shown in (B) and (C) because the ARTIC primer sets used in COG-UK sequencing changed from version 3 (B) to version 4 (C). This resulted in the periodic recurrences of variation frequencies because of Illumina sequencing error rates being different between the two approaches. We note that for both primer sets the sequencing error rates increased towards the end of each read and as a result would cause the average variation frequency of each amino acid to peak in the middle of the amplicon.


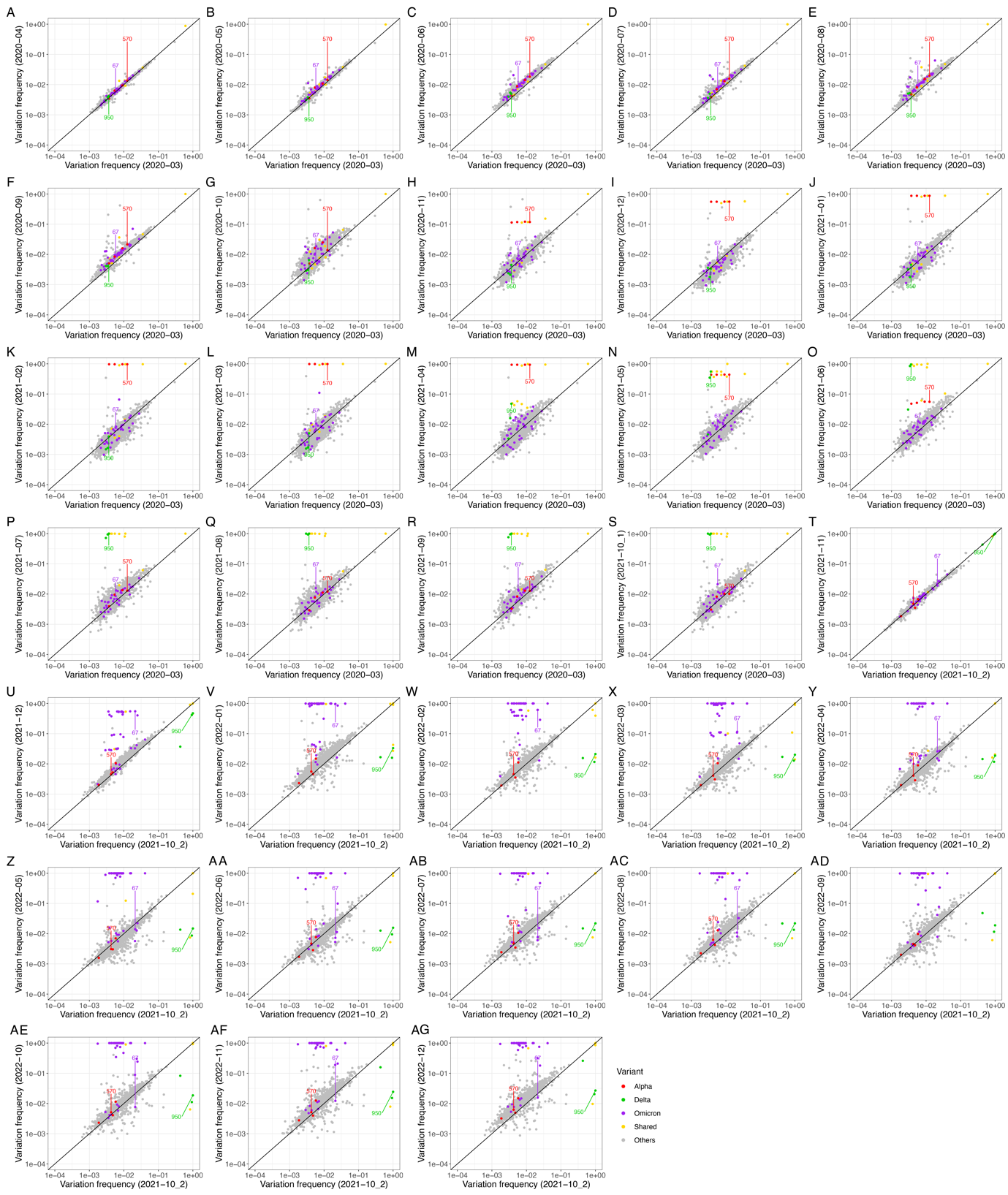


Supplementary Figure S2. (A-S) Monthly average variation frequency of each amino acid site in spike protein from April 2020 to October (1^st^ to 15^th^) 2021 was plotted against that of March 2020. (T-AG) Monthly average variation frequency of each amino acid site in spike protein from November 2021 to December 2022 was plotted against to that of October (16^th^ to 31^st^) 2021, while the ARTIC primer sets used in COG-UK sequencing changed from version 3 to version 4. “shared” indicates the amino acid mutation was shared by different variants. For example, as referred to in the main text, the spike:D614G substitution is shown and maintained in the top right corner. Colored dots indicated in the key are amino acids associated with the respective VoC (red linked to Alpha, green linked to Delta and purple linked to Omicron). Amino acids that are shared by the different VoCs are indicated in yellow. The location of one amino acid of each Vocs in each protein was labelled.


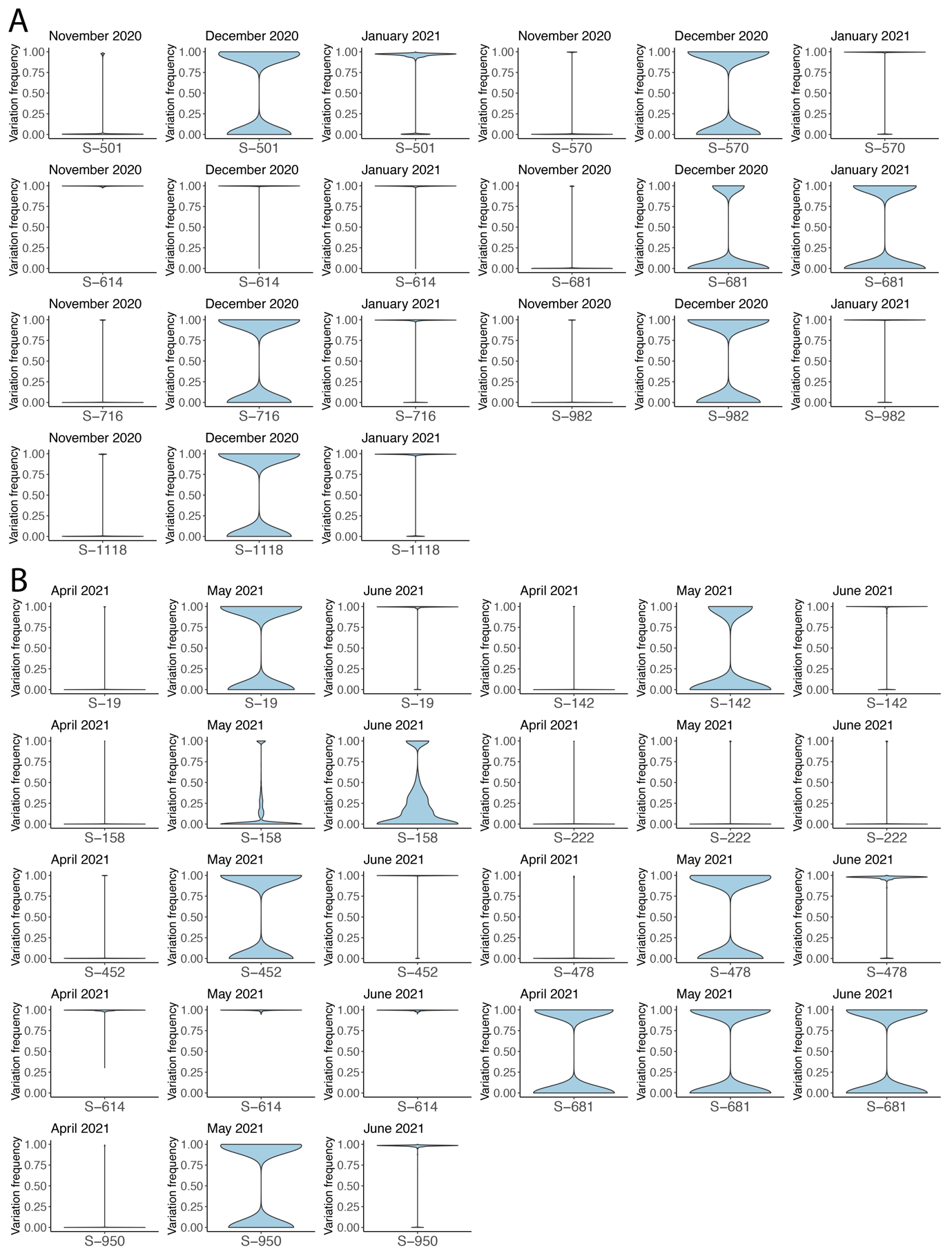


Supplementary Figure S3. (A) The transition of N501Y, A570D, D614G, P681H, T716I, S982A and D1118H of Alpha variant and (B) T19R, G142D, R158G, A222V, L452R, T478K, D614G, P681R and D950N of Delta variant on spike protein over time in the minor genomic variants. The width of the violin plot indicates the number of samples with the frequency on the y-axis. Samples with variation frequency = 1.00 indicates complete transition of the mutation.


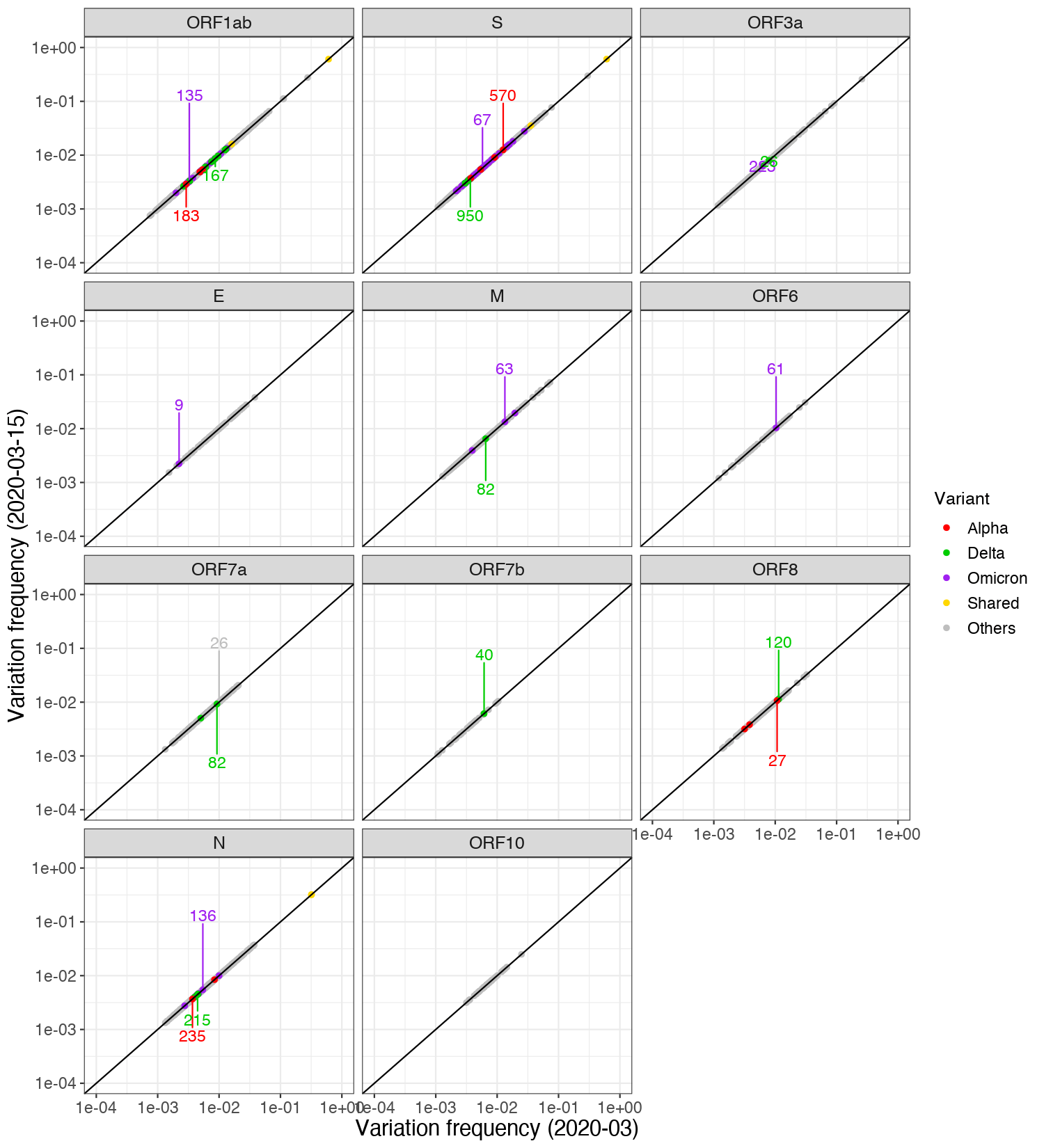


Supplementary Figure S4. Animation of all gene data of (A-S) monthly average variation frequency of each amino acid site from April 2020 to October (1^st^ to 15^th^) 2021 was plotted against that of March 2020, and (T-AG) monthly average variation frequency of each amino acid site from November 2021 to December 2022 was plotted against to that of October (16^th^ to 31^st^) 2021, while the ARTIC primer sets used in COG-UK sequencing changed from version 3 to version 4. “shared” indicates the amino acid mutation was shared by different variants. Please click “play” on the figure to watch the animation. Colored dots indicated in the key are amino acids associated are associated with the respective VoC. Amino acids that are shared by the different VoCs are indicated in yellow. The location of one amino acid of each Vocs in each protein was ladled.


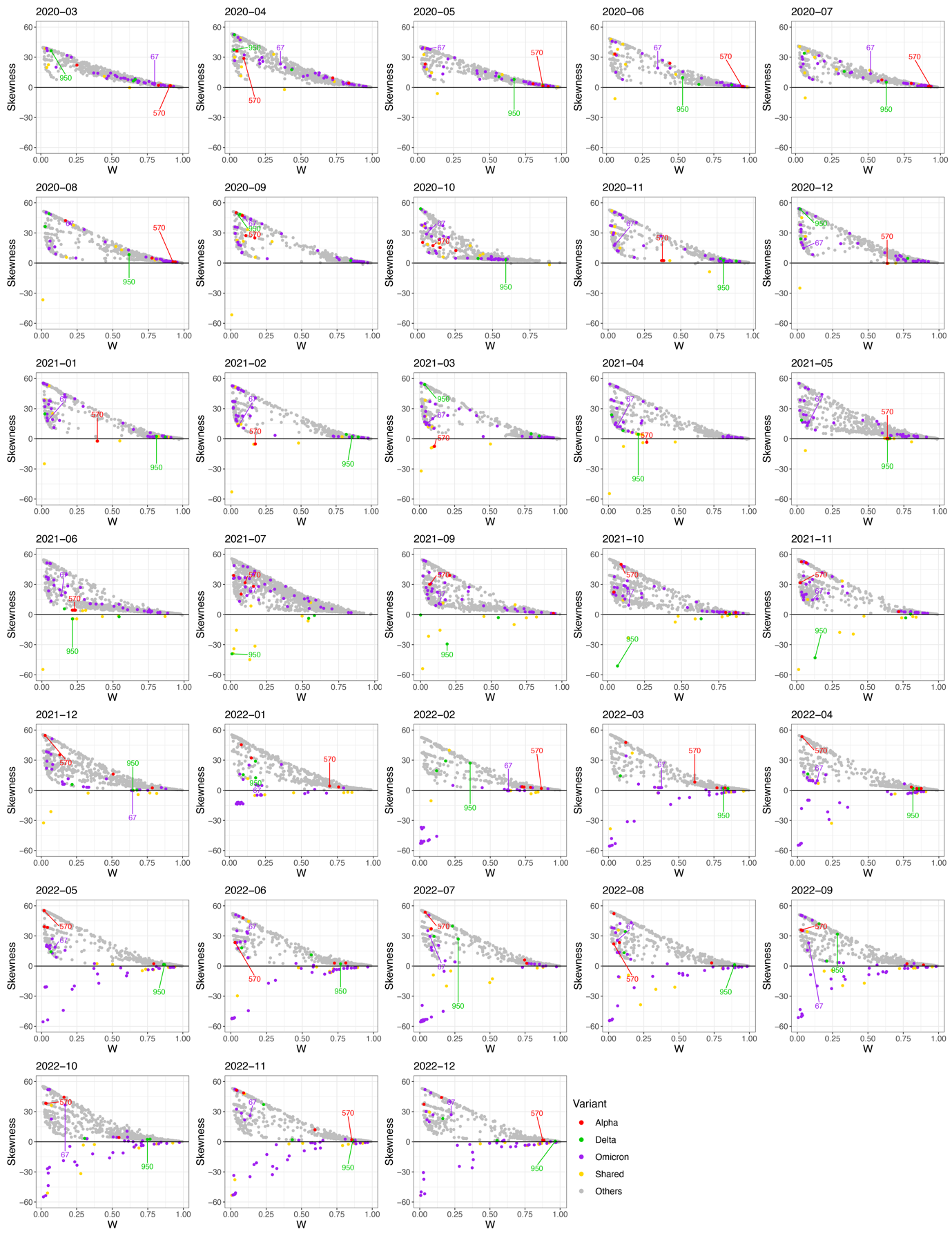


Supplementary Figure S5. Skewness value (y-axis) plotted against W value (x-axis) for each amino site in each month. Individual amino acids are shown by a dot. A positive skewness value indicates a right-skewed distribution, while a negative skewness value indicates a left-skewed distribution. Colored dots indicated in the key are amino acids associated are associated with the respective VoC. Amino acids that are shared by the different VoCs are indicated in yellow. Colored dots indicated in the key are amino acids associated with the respective VoC (red linked to Alpha, green linked to Delta and purple linked to Omicron). Amino acids that are shared by the different VoCs are indicated in yellow. The location of one amino acid of each Vocs in each protein was labelled.


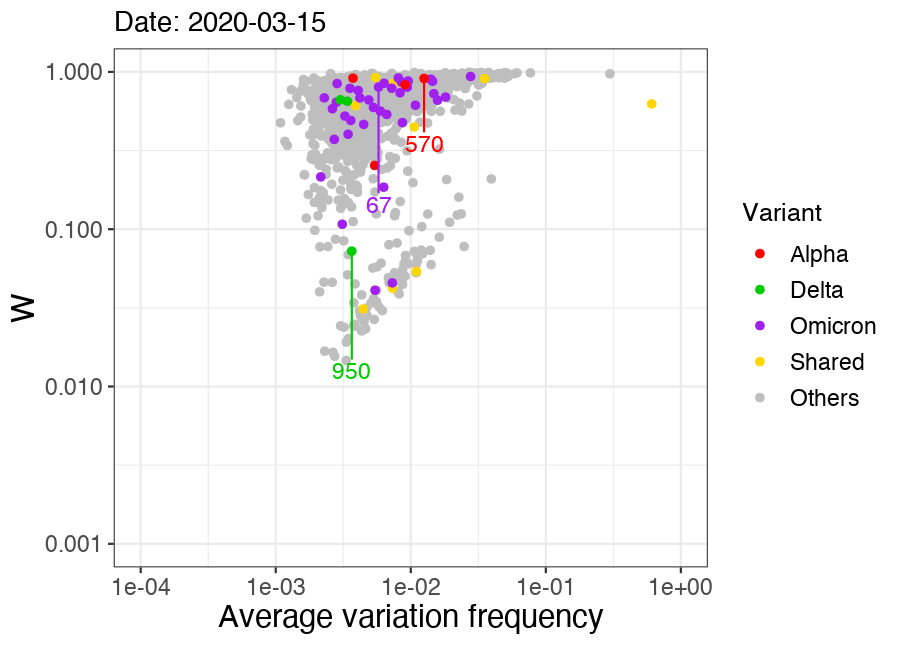


Supplementary Figure S6. Animation and sound file of the data shown in Supplementary Figure S7. For the animation the average variation frequency is shown on the x axis and the W value on the Y axis. These values are shown over the sampling period (March 2020 to December 2022). Each dot represents an individual amino acid on the spike protein with those amino acids that form part of VoCs coloured as described in the key. The Supplementary Sound (Mp4 of Dong’s sonata of SARS2 Spike) is an interpretation of the W value by converting the data into music with Python (https://github.com/SYSTEMSounds/sonification-tutorials). Inspiration for this was taken from Project Hail Mary by Andy Weir where Rocky sees in sound. Please click “play” on the figure to watch the animation. Colored dots indicated in the key are amino acids associated with the respective VoC (red linked to Alpha, green linked to Delta and purple linked to Omicron). Amino acids that are shared by the different VoCs are indicated in yellow. The location of one amino acid of each Vocs in each protein was labelled.


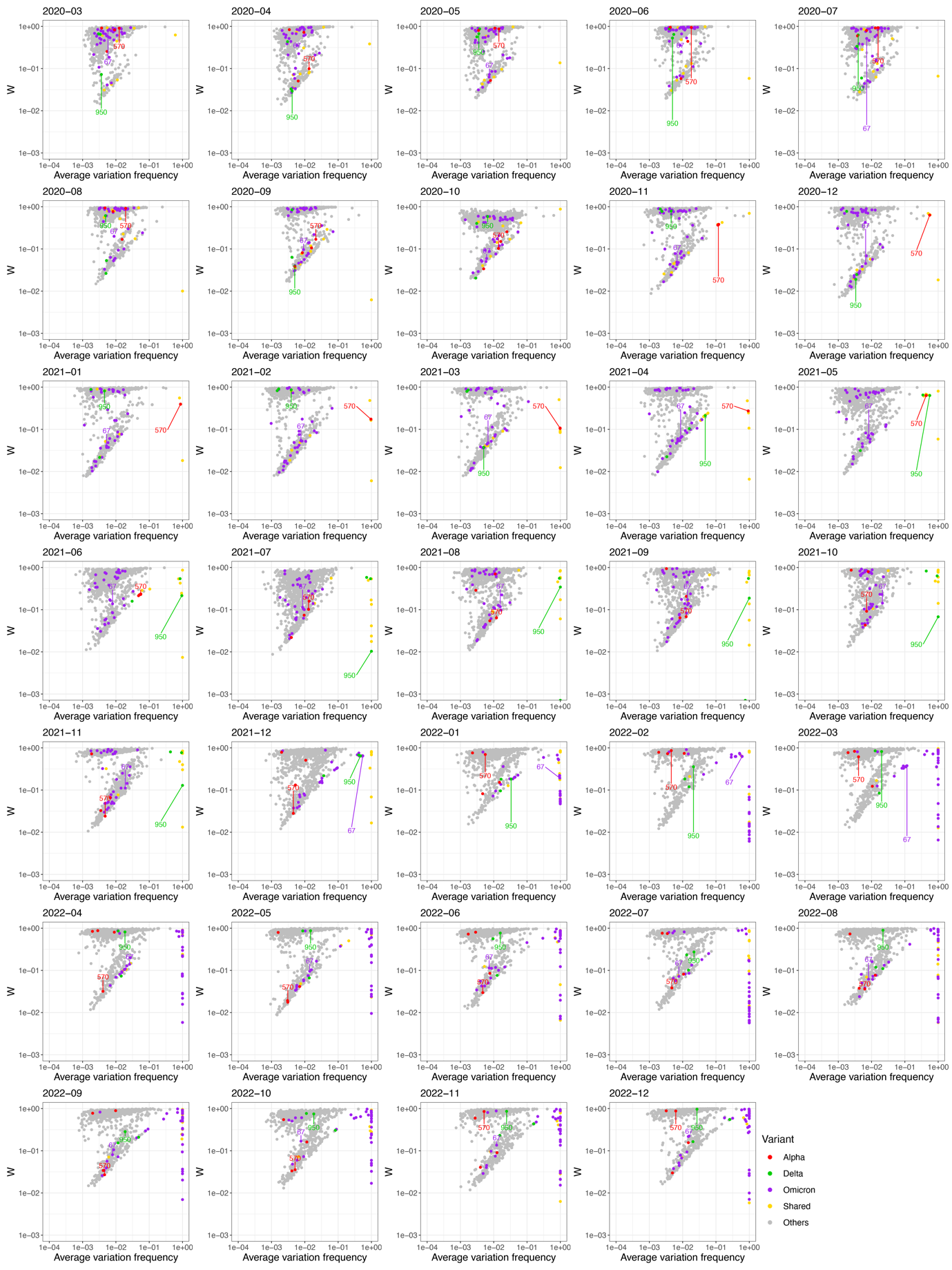


Supplementary Figure S7. W value plotted against average variation frequency for each amino site in each month. Colored dots indicated in the key are amino acids associated with the respective VoC (red linked to Alpha, green linked to Delta and purple linked to Omicron). Amino acids that are shared by the different VoCs are indicated in yellow. The location of one amino acid of each Vocs in each protein was labelled.


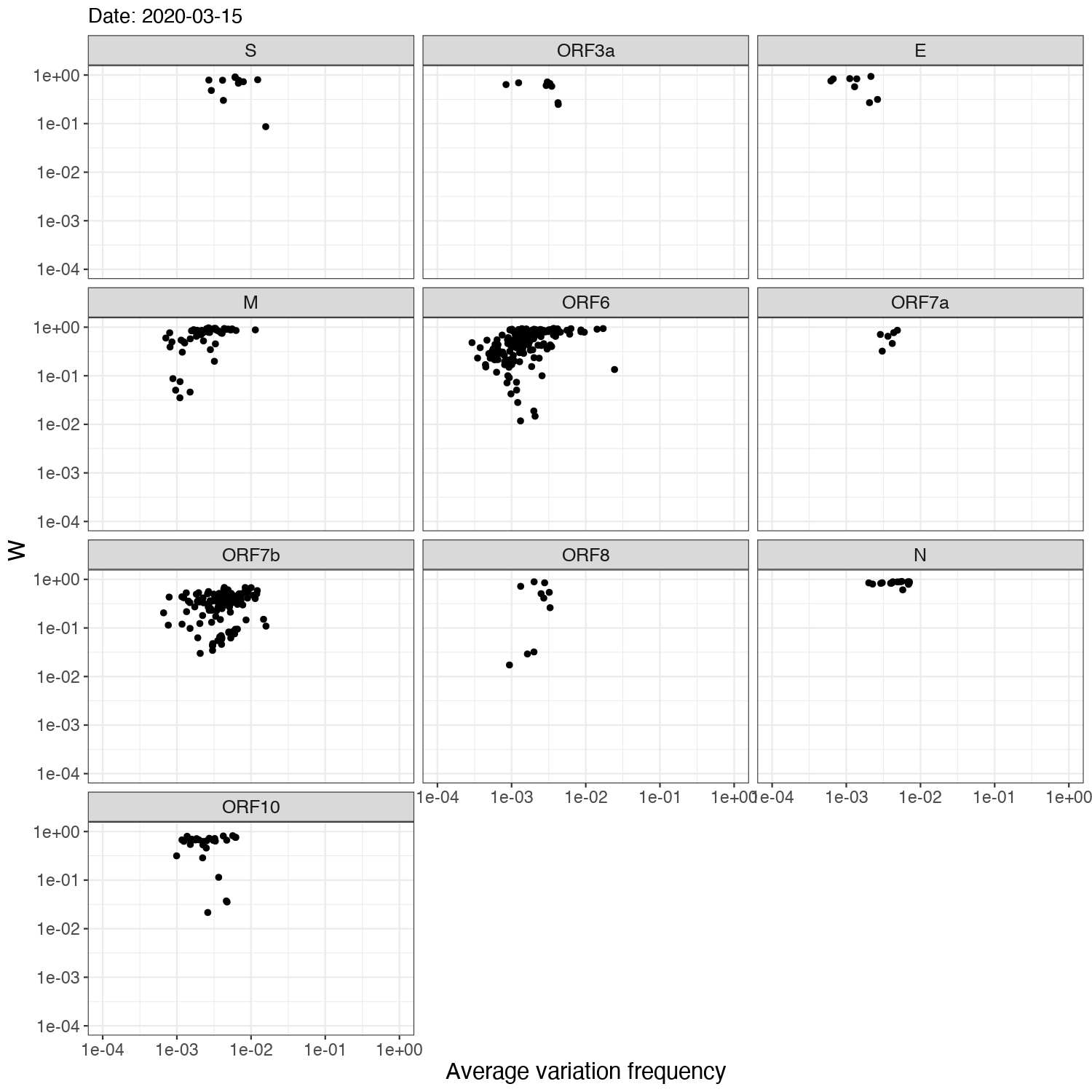


Supplementary Figure S8. Animation of W value plotted against average variation frequency for each nucleotide sites of TRS region of each sub-genome in each month. Please click “play” on the figure to watch the animation.


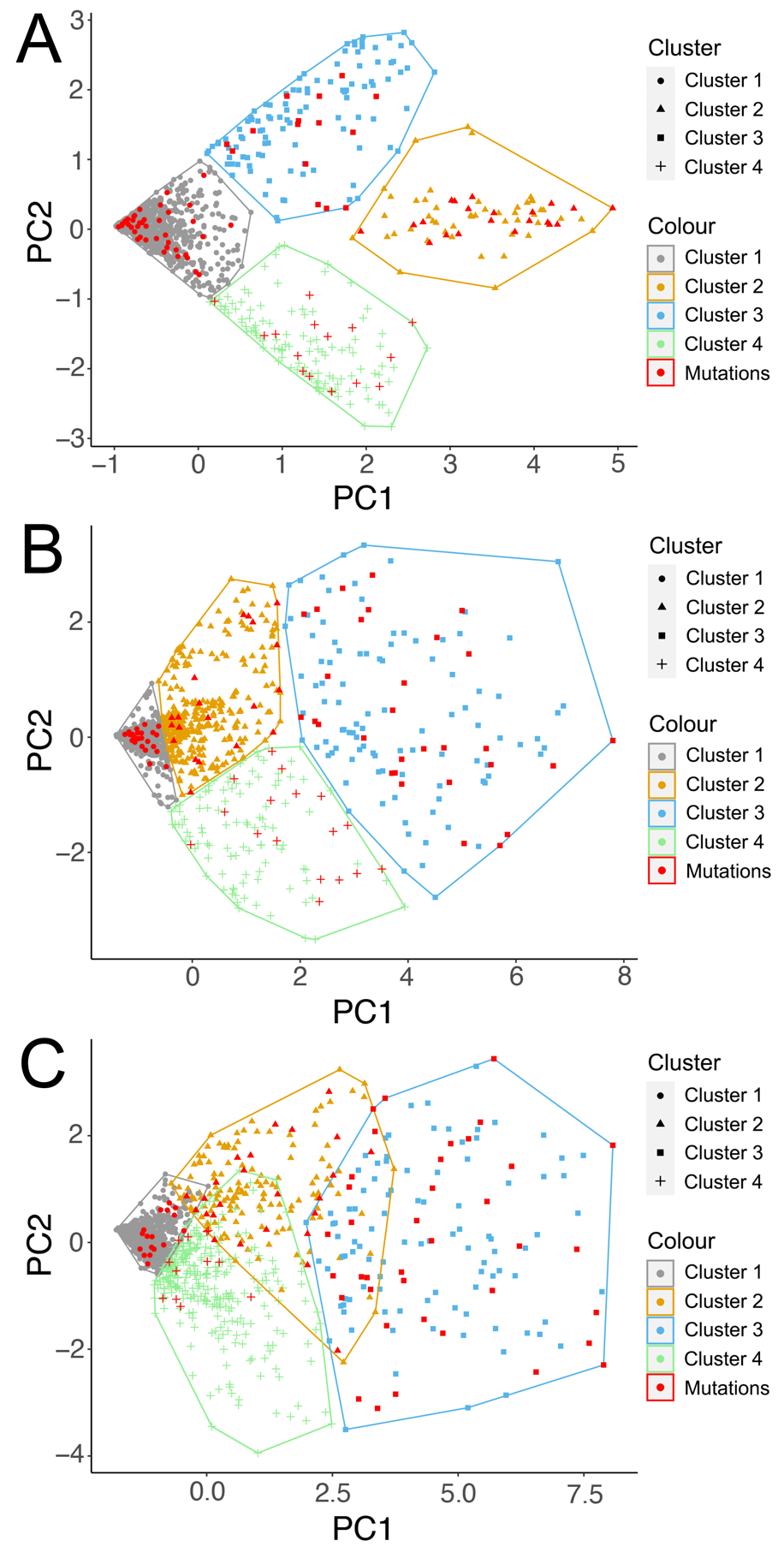


Supplementary Figure S9. Scatter plots of pam clusters with colouring and shaping each data point according to its cluster assignment for (A) March 2020 to May 2020 (pam3), (B) March 2020 to August 2022 (pam6) and (C) March 2020 to November 2022 (pam9). Substitution sites used for test were coloured in red.

Supplementary Figure S10. W value (y-axis) of each amino acid site (x-axis) from N-terminal to C-terminal end along the spike protein by month and year of the COVID-19 pandemic indicated to the top left.


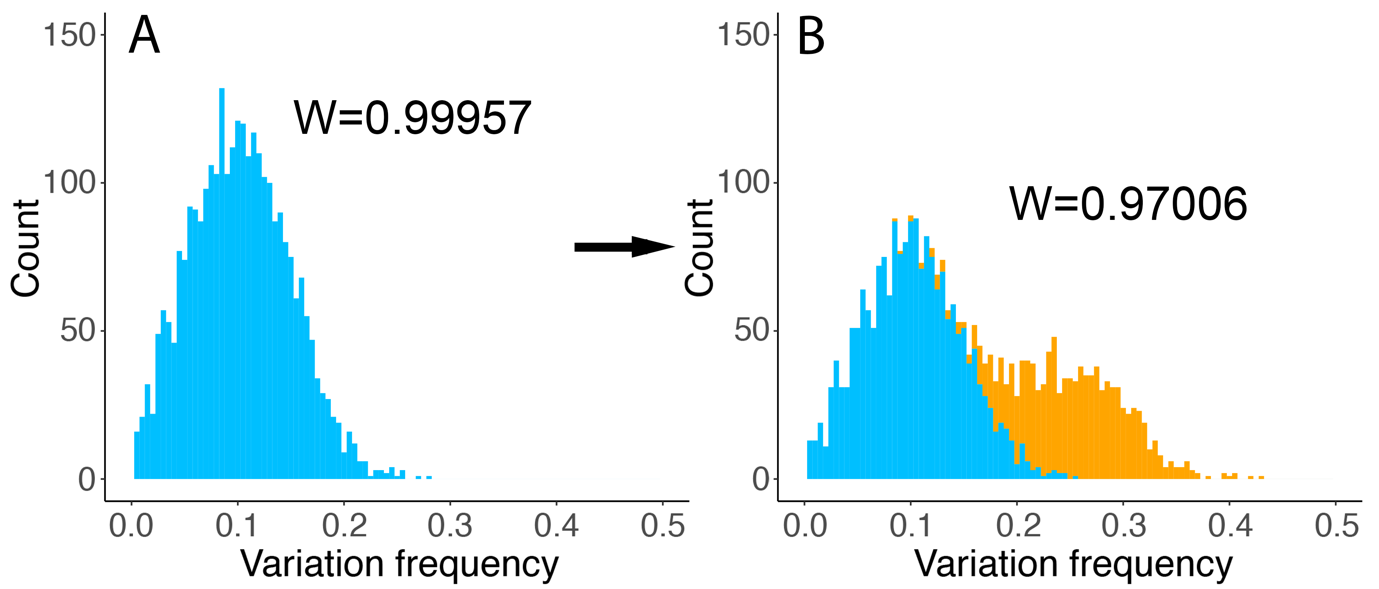


Supplementary Figure S11. A simulation of the transition from a normal distribution of variation frequencies at an amino acid site (A) to skewed distributions (B) in 3,000 samples. Blue bars show the number of samples with sequencing error only, and orange bars indicated samples with minor variation.
