# Supplementary material for: Using minor variant genomes and machine learning to study the genome biology of SARS-CoV-2 over time
Source: Nucleic Acids Res. 2025 Feb 19;53(4):gkaf077. doi: 10.1093/nar/gkaf077 (PMC11838042; doi:10.1093/nar/gkaf077)
Supplement: gkaf077_Supplemental_Files [file gkaf077_supplemental_files.zip › Supplementary_Materials.docx]

**Supplementary Materials**

**Commentary on ARTIC primer sets and versions and variability.**

To investigate whether mutable sites in the SARS-CoV-2 genome could be identified we studied viral population genetics over the course of nearly three years and coupled this with a machine learning approach. The average variation frequencies of each amino acid position along the viral genome/proteome were calculated to investigate the emergence of substitutions in Spike over the three years sampled. Amplicon sequencing could introduce low-frequency genetic variants due to errors acquired during PCR/RT-PCR and Illumina sequencing (1). These low-frequency genetic variants may result in overestimation of amino acid variation frequency. The average variation frequencies of each amino acid were plotted in Spike by month in 2020, 2021 and 2022, respectively (Supplementary Figure S1B and C). The amino acid variation frequencies showed periodic recurrence (Supplementary Figure S1B and C). Each wave is situated between a paired primer pair and peaked in the middle of the amplicon (Additional file 1: Fig. S1B and C). For COG-UK SARS-CoV-2 genomes were amplified using ARTIC primers version 3 from March 2020 to 15th October 2021 and ARTIC primers version 4 from 16th October 2020 to 31st December 2022. ARTIC primers versions 3 and 4 amplify different regions of the SARS-CoV-2 genome that again corresponded to period recurrence of amino acid variation frequencies in the Spike protein (Supplementary Figure S1B and C). Hence, these periodic recurrences were likely a result of Illumina associated sequencing error. even though our analysis pipeline had included an Illumina sequencing error reduction algorithm (see method). ARTIC primers produced amplicons of approximately 390 nucleotides. Illumina sequenced each amplicon from both sides, resulting in read lengths of 220 bp for R1 and 200 bp for R2 in the COG-UK datasets. Illumina sequencing error rates increased towards the end of each read (2) and would cause the average variation frequencies of each amino acid to peak in the middle of the amplicon (intersection of R1 and R2 ends). Although the overall error rates of Illumina sequencing were very low, the sequencing error would accumulate at same places in the amplicons (approximately 0.06 in the peak region as showed on Supplementary Figure S1B and C) because of repeated sequencing of same amplicon. The average variation frequencies of each amino acid showed the same periodic error pattern in different months (Supplementary Figure S1B and C) and indicated the Illumina sequencing and PCR/RT-PCR error rates were almost identical with same ARTIC primers version at same nucleotide site and subsequent amino acid site in different samples. Therefore, if the samples have been amplified by the same ARTIC primers version, comparing the average variation frequencies of amino acids at the same site in different months would reveal real differences in frequencies of amino acid changes. Periodic recurrence, as shown in Supplementary Figure S1B and C, was not observed in the average variation frequencies of W values (Supplementary Figure S10). Therefore, the W value is relatively unbiased and can serve as a reliable variable for machine learning. Samples with a normal distribution of error will result in W with a high value and samples deviating away from a normal distribution will have a lower value of W. As discussed above, the Illumina sequencing and PCR/RT-PCR error rates are almost identical at the same amino acid site in different samples. We analyzed about 3,000 independent samples per month and measured the variation frequency at each amino acid site across the viral genome/proteome. Based on the Central Limit Theorem (3), if the error rate does not change and the number of independent measurements increases, the probability density of the measured error rates tends to follow a normal distribution with a mean close to the true error rate.

**Commentary on W value usage**

**Illumina sequencing error in the ideal world.** If a nucleotide/amino acid site in all virus genomes in a sample is identical to the site on the original virus genome (Wuhan strain), meaning this nucleotide/amino acid site was not subjected to any evolutionary forces (selection and stochastic evolutionary processes) and remained unchanged in all virus genomes in a sample, the real variation frequency would be 0. However, due to the Illumina sequencing error, the measured variation frequency (measurement error) of this nucleotide/amino acid site would be above 0.

Based on the Central Limit Theorem, which states that the sum of a large number (this is our 3,000 patients per month) of independent random variables is approximately normally distributed, measurement errors tend to follow a normal distribution (4-6). In this study, the Illumina sequencing approach was used to determine the nucleotide base in the virus genome at each position. Measurement errors of Illumina sequencing at each nucleotide/amino acid site can be caused by a variety of different factors, such as crosstalk, phasing, fading or T accumulation (7), and could be qualified using variation frequency. We investigated the samples by month, and approximately 3,000 samples were randomly collected in each month from the COG UK data sets. In other words, a specific nucleotide/amino acid site was independently measured 3,000 times. If we assume this nucleotide/amino acid site was not subjected to any evolutionary forces in all 3,000 samples, due to the large number of independent measurements, the distribution of the measured variation frequencies (measurement errors) of these 3,000 samples would approximately follow a normal distribution.

**Nucleotide/amino acid site** **variation in the real world.** Mutation events occurring during virus replication would provide new genetic resources, first at a minor variant genome level. If these new substitutions at an amino acid site enable the virus to adapt better to grow in the host, or transmit between hosts, they may be transmitted to other individuals as part of the infecting viral swarm and probably become dominant in downstream infected individuals (8). If amino acid substitutions are neutral, the amino acid frequency changes are likely due to nondirectional stochastic evolutionary processes. If amino acid substitutions are non-neutral, the amino acid frequencies can be decided by directional positive and purifying selection. Either/both positive selection or/and stochastic evolutionary processes could result in a change in amino acid frequency. Therefore, an amino acid site under evolutionary forces, especially natural selection, would result in increased frequencies of variations at this amino acid site in some of these 3,000 samples to a minor level (some virus genomes in an individual have this mutation), or to dominant levels (most virus genomes in an individual have this mutation). This disruption would deviate from the normal distribution of variation frequencies at the amino acid site, causing skewed distributions and reducing the W value (see main text). However, if substitutions develop at an amino acid site that are harmful to the virus, they would be quickly eliminated, and the variation frequency at this amino acid site would not be significantly raised. Consequently, the variation frequencies, caused only by Illumina sequencing error, at this site would tend toward a normal distribution (W tends to 1).

In the main text, we discussed how the transition from a normal distribution of variation frequencies at an amino acid to skewed distributions could reduce the W value. Here, to illustrate this concept, a simulation was employed to illustrate the process of a transition due to minor genomic variation and the corresponding change of W value in 3,000 samples. Firstly, a normal distribution of sequencing error was simulated, with a mean of 0.1 and with a standard deviation of 0.1 and a resulting W value of 0.99957 (Supplementary Figure S11A). This shows that under no evolutionary forces the W value tends towards 1. Next the simulation was run with the assumption that 1000 (random) of these 3,000 samples had a minor variation frequency of 0.15 that were added to the background sequencing error (orange bars showed in Supplementary Figure S11B). The number of samples with sequencing error only (blue bars) was reduced (but still had a normal distribution) whereas the plot of total samples was right skewed due to the samples with minor variation plus the same sequencing error (orange and green bars). This resulted in an overall reduction in the W value to 0.97006, and thus indicated that some of the population was under evolutionary pressure.

**Variation frequency and** **W value in the data we analyzed.** Through observation of the W value-average variation frequency animation plot shown in Supplementary Figure S6, we identified two distinct major clusters of amino acid sites in Spike throughout all the months analyzed (Supplementary Figure S6 and S7 and Figure 3B). As discussed in the main text some amino acid sites in Spike maintained high W values close to 1 throughout all three years of the pandemic sampled, while others present in the low W value or shifted between high and low W value clusters (Supplementary Figures S6 and S7). All amino acid substitutions that defined the VoCs travelled through the cluster associated with a low W value before they became dominant in the population (Supplementary Figure S6 and S7). We found that amino acid sites associated with the VoCs were present in the low W value cluster or shifted between high and low W value clusters (under evolutionary pressure) from the start of the pandemic (Supplementary Figures S6 and S7). Especially, substitutions associated with Omicron were observed in the low W value cluster or shifted between high and low W value clusters in the first three months of the UK data analyzed in 2020. Indeed, we have found amino acid substitutions belonging to VoCs, including Omicron, were already present as minor genomic variants in the early cases from the Huanan Market at the beginning of the pandemic in 2019 (9).

**Usage of W value in unsupervised machine learning.** An unsupervised machine learning approach can partition data into distinct groups of similar items. These amino acids in Spike, kept in the cluster of high W values close to 1 throughout all three years of the pandemic and unlikely to stay in the low W value cluster or shift between high and low W value clusters (Supplementary Figures S6 and S7), could be partitioned into the same cluster with the unsupervised machine learning approach. Amino acid sites that have been conserved in the past 34 months are likely to be conserved for a significant period in the future, as no substitutions caused by the vaccine have been reported (10) and there are unlikely to be many additional evolutionary forces acting on these sites at a population level. The cluster 1s in Figure 4A and B, and Supplementary Figure S9A, B, and C showed the highest average W value (Table 1). Therefore, these sites within the cluster 1s were considered as conserved amino acid sites, while the sites outside the cluster 1s were considered as mutable amino acid sites. To evaluate the performance of the clustering of the conserved sites in the unsupervised machine learning analysis, the silhouette coefficient was derived (11). This coefficient ranges from -1 to 1 and is considered good evidence for clustering if it exceeds 0.5, or as strong evidence if it exceeds 0.7 (12,13). The average silhouette score for the conserved cluster (cluster 1) in pam12 was 0.691 in pam12 and 0.636 in pam34 (Supplementary Table S10).

**Reference**

1. Guenay-Greunke, Y., Bohan, D.A., Traugott, M. and Wallinger, C. (2021) Handling of targeted amplicon sequencing data focusing on index hopping and demultiplexing using a nested metabarcoding approach in ecology. *Scientific reports*, **11**, 19510.

2. Schirmer, M., D’Amore, R., Ijaz, U.Z., Hall, N. and Quince, C. (2016) Illumina error profiles: resolving fine-scale variation in metagenomic sequencing data. *BMC bioinformatics*, **17**, 1-15.

3. Kwak, S.G. and Kim, J.H. (2017) Central limit theorem: the cornerstone of modern statistics. *Korean journal of anesthesiology*, **70**, 144-156.

4. Ross, S.M. (2017) *Introductory statistics*. Academic Press.

5. Pishro-Nik, H. (2014) *Introduction to probability, statistics, and random processes*. Kappa Research, LLC Blue Bell, PA, USA.

6. Gierlinski, M. (2015) *Understanding statistical error: a primer for biologists*. John Wiley & Sons.

7. Nakamura, K., Oshima, T., Morimoto, T., Ikeda, S., Yoshikawa, H., Shiwa, Y., Ishikawa, S., Linak, M.C., Hirai, A. and Takahashi, H. (2011) Sequence-specific error profile of Illumina sequencers. *Nucleic acids research*, **39**, e90-e90.

8. Goldswain, H., Dong, X., Penrice-Randal, R., Alruwaili, M., Shawli, G.T., Prince, T., Williamson, M.K., Raghwani, J., Randle, N. and Jones, B. (2023) The P323L substitution in the SARS-CoV-2 polymerase (NSP12) confers a selective advantage during infection. *Genome biology*, **24**, 47.

9. Dong, X. and Hiscox, J.A. (2023) Analysis of SARS-CoV-2 Population Genetics from Samples Associated with Huanan Market and Early Cases Identifies Substitutions Associated with Future Variants of Concern. *Viruses*, **15**, 1728.

10. Gu, H., Quadeer, A.A., Krishnan, P., Ng, D.Y., Chang, L.D., Liu, G.Y., Cheng, S.M., Lam, T.T., Peiris, M. and McKay, M.R. (2023) Within-host genetic diversity of SARS-CoV-2 lineages in unvaccinated and vaccinated individuals. *Nature Communications*, **14**, 1793.

11. Müller, A.C. and Guido, S. (2016) *Introduction to machine learning with Python: a guide for data scientists*. " O'Reilly Media, Inc.".

12. Dalmaijer, E.S., Nord, C.L. and Astle, D.E. (2022) Statistical power for cluster analysis. *BMC bioinformatics*, **23**, 1-28.

13. Kaufman, L. (1990) Partitioning around medoids (program pam). *Finding groups in data*, **344**, 68-125.
